# Spatiotemporal perturbations of the plasminogen activation system in a rat model of acute organophosphate intoxication

**DOI:** 10.1186/s40478-025-01979-0

**Published:** 2025-03-18

**Authors:** Thomas J. Blackmon, Jeremy A. MacMahon, Pedro N. Bernardino, Ryan E. Hogans, Mei-Yun Cheng, Joan Vu, Ruth Diana Lee, Naomi H. Saito, Ana Cristina Grodzki, Donald A. Bruun, Heike Wulff, Kevin D. Woolard, Amy Brooks-Kayal, Danielle J. Harvey, Fredric A. Gorin, Pamela J. Lein

**Affiliations:** 1 Department of Molecular Biosciences, School of Veterinary Medicine, University of California, Davis, CA 95616 USA; 2 Department of Pharmacology, School of Medicine, University of California, Davis, CA 95616 USA; 3 Department of Public Health Sciences, School of Medicine, University of California, Davis, CA 95616 USA; 4 Department of Pathology, Microbiology, and Immunology, School of Veterinary Medicine, University of California, Davis, CA 95616 USA; 5 Department of Neurology, School of Medicine, University of California, Davis, Sacramento, CA 95817 USA; 6 Molecular Biosciences, UC Davis School of Veterinary Medicine, 1089 Veterinary Research Drive, Davis, CA 95616 USA

**Keywords:** Blood-brain barrier, Diisopropylfluorophosphate, Epilepsy, Neuroinflammation, Plasminogen activator inhibitor-1 (PAI-1)

## Abstract

Neuroinflammation is widely posited to be a key pathogenic mechanism linking acute organophosphate (OP)-induced *status epilepticus* (SE) to persistent brain injury and abnormal electrical activity that contribute to epilepsy and cognitive impairment. The plasminogen activation system (PAS) promotes neuroinflammation in diverse neurological diseases but whether it is activated following acute OP intoxication has yet to be evaluated. To address this data gap, we characterized the spatiotemporal expression patterns of multiple components of the PAS in a rat model of acute intoxication with the OP, diisopropylfluorophosphate (DFP). Adult male Sprague Dawley rats administered DFP (4 mg/kg, sc), atropine sulfate (2 mg/kg, im) and 2-pralidoxime (25 mg/kg, im) went into SE that persisted for hours. One day after acute DFP-induced SE, plasmin activity and protein concentrations of plasminogen activator inhibitor-1 (PAI-1) in the plasma were increased, though not significantly. In contrast, acute DFP intoxication significantly increased brain levels of PAI-1, tissue-type plasminogen activator (tPA), urokinase plasminogen activator (uPA), and transcripts of TGF-β in a time- and region-dependent manner. In the cortex and hippocampus, quantification of PAI-1, tPA, and uPA by ELISA indicated significantly increased levels at 1 day post-exposure (DPE). PAI-1 and uPA returned to control values by 7 DPE while tPA protein remained elevated at 28 DPE. Immunohistochemistry detected elevated PAI-1 expression in the DFP brain up to 28 DPE. Co-localization of PAI-1 with biomarkers of neurons, microglia, and astrocytes demonstrated that PAI-1 localized predominantly to a subpopulation of astrocytes. Cytologically, PAI-1 localized to astrocytic end feet, but not adjacent neurovascular endothelium. Electron microscopy revealed neuronal metabolic stress and neurodegeneration with disruption of adjacent neurovascular units in the hippocampus post-DFP exposure. These data indicate that acute DFP intoxication altered PAS expression in the brain, with aberrant PAI-1 expression in a subset of reactive astrocyte populations.

**Supplementary Information:**

The online version contains supplementary material available at 10.1186/s40478-025-01979-0.

## Introduction

Organophosphate (OP) poisoning is associated with cholinergic crisis, a toxidrome caused by acute inhibition of synaptic acetylcholinesterase by > 60–70%, which triggers life-threatening parasympathomimetic symptoms, *status epilepticus* (SE) and cardiopulmonary arrest [1, 2]. Persistent neurological morbidities have been documented in humans who survive acute OP intoxication, including acquired epilepsy [3], cognitive and motor impairments [4, 5], and altered personality [6, 7]. Similar long-term adverse neurological effects [8–10] are observed in animal models of acute OP intoxication [11]. For example, acute intoxication of rats with the OP diisopropylfluorophosphate (DFP) induces refractory SE with subsequent neurodegeneration, spontaneous recurrent seizures (SRS), cognitive deficits and persistent brain injury [8, 12–15]. While current standard of care (SOC) for acute OP intoxication improves survival, there remain critical unmet needs for: (1) therapeutic strategies that can be administered following the acute response phase to protect against SRS and cognitive dysfunction associated with OP-induced cholinergic crisis; and (2) biomarkers to identify survivors at increased risk for chronic, adverse neurological effects who may benefit most from adjunct therapies. Efforts to address these needs have been stymied by lack of understanding of pathogenic mechanism(s) linking the acute to the chronic neurotoxic effects of acute OP intoxication.

We and others have observed that acute DFP intoxication in the rat is associated with a robust neuroinflammatory response, including microglial activation and astrogliosis that persists for at least 6 months post-exposure [12, 16, 17]. Whether persistent neuroinflammation similarly occurs in the human brain following acute OP intoxication is unknown; however, human necropsy studies have shown that acute OP poisoning is associated with inflammation of cardiac and pancreatic tissues [18]. Moreover, recent epidemiological reports have identified an increased incidence of deep vein thromboses and recurrent seizures in human survivors of acute OP poisoning. Such observations suggest that acute OP intoxication may dysregulate the plasminogen activation system (PAS) [3, 19].

The canonical function of the PAS is modulation of fibrinolysis. The zymogen plasminogen (Plg) is converted to active plasmin by urokinase-type plasminogen activator (uPA) and tissue-type plasminogen activator (tPA); activated plasmin degrades fibrin deposits to breakdown blood clots. tPA, which is the primary plasmin activator involved in intravascular fibrinolysis, is irreversibly inactivated when complexed with PAI-1 [20]. In addition to its role in fibrinolysis, the PAS promotes inflammatory responses [21, 22], including neuroinflammatory responses in the brain, and modulates blood-brain barrier (BBB) permeability [20, 23, 24]. tPA has been shown to cross the BBB, alter the neurovascular unit (NVU) and cause BBB dysfunction via plasmin-dependent and plasmin-independent mechanisms [20]. PAI-1 is an acute phase pro-inflammatory reactant that enables neutrophil infiltration of microvascular endothelial cells during reperfusion-injury, causing vascular leakage and the secretion of cytokines that promote neuroinflammation [25, 26].

**Fig. 1:**
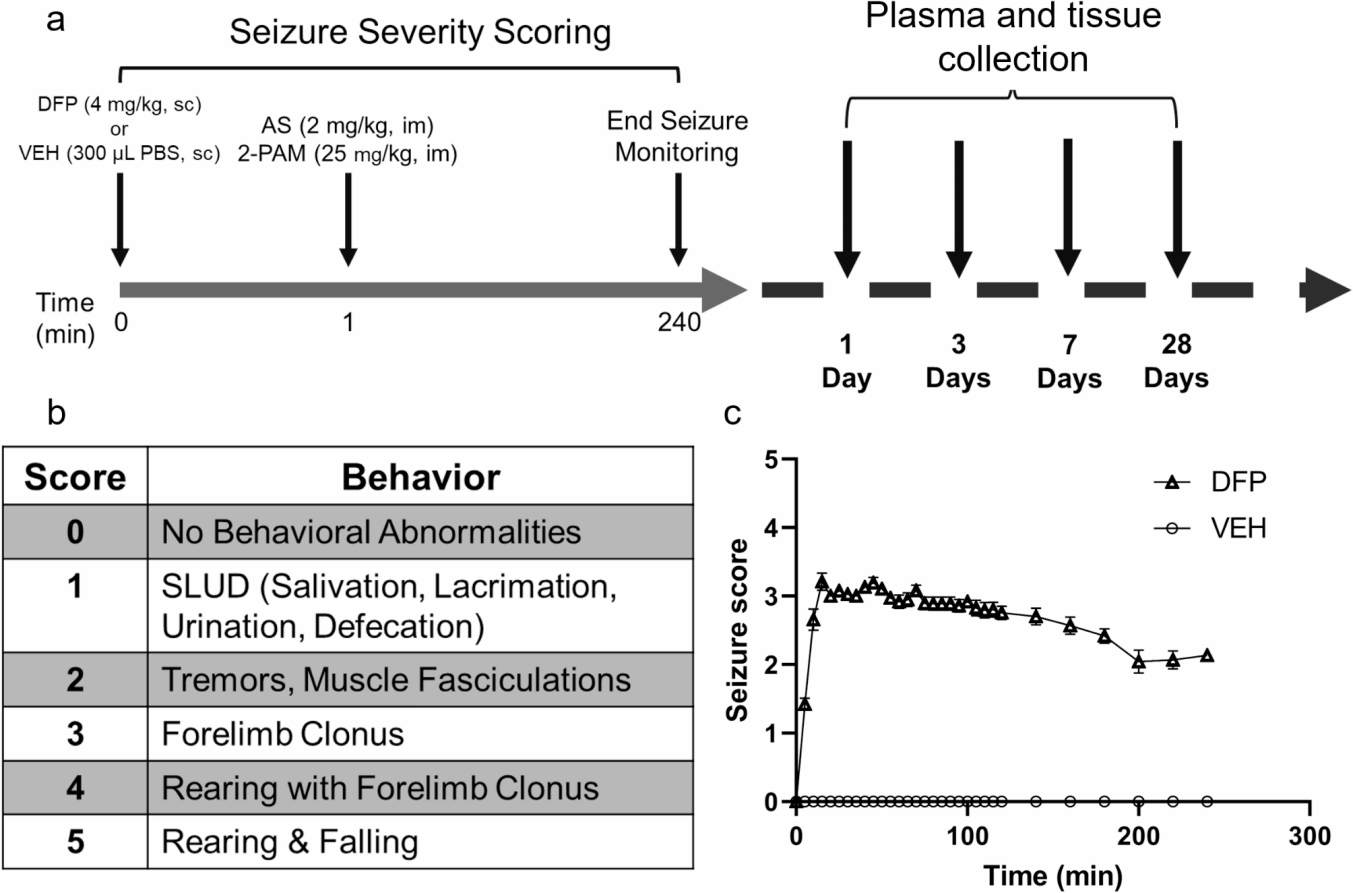
Rat model of acute diisopropylflurophosphate (DFP) intoxication (**a**) Schematic of the dosing paradigm used to initiate DFP-induced seizures in adult male Sprague Dawley rats and timeline of sample collection. (**b**) Behavioral seizure scale used to score seizure behavior in DFP-intoxicated rats. (**c**) Profile of seizure scores in DFP and vehicle (VEH) animals. Data presented as mean ± SE (*n* = 38 DFP and 19 VEH)

Decreased tPA and increased PAI-1 in hippocampus have been reported in mice between 3 h and 3 d after SE induced by pilocarpine, a muscarinic receptor agonist, and these changes are associated with reduced pro-BDNF cleavage [27]. To our knowledge, the impact on the PAS of acute intoxication with OP cholinesterase inhibitors has not been previously investigated. While the acute SE induced by pilocarpine and OPs are similar electrophysiologically, the cholinergic receptors that mediate seizure activity and the profile of chronic neurological effects elicited by these two convulsant chemotypes differ [28]; therefore, the response of the PAS to acute OP intoxication cannot be inferred by studies conducted in models of acute pilocarpine-induced SE. Here, we employed a well-characterized rat model of acute DFP intoxication (Fig. 1a) to test the hypothesis that acute OP intoxication alters the PAS. We found that PAI-1 levels in the plasma and discrete regions of the brain were elevated immediately following acute DFP-induced SE. Plasmin activator levels were also elevated in the hippocampus and cortex, and expression of PAI-1 within distal astrocytic processes and end feet progressively increased with increasing time post-exposure.

## Materials and methods

### Animals and exposure

Animals were maintained in facilities fully accredited by the Association for Assessment and Accreditation of Laboratory Animal Care (AAALAC), and all studies were performed with regard to alleviating pain and suffering under protocols approved by the UC Davis Institutional Animal Care and Use Committee (IACUC protocol numbers 201865 and 201954). Animal experiments were conducted and reported in accordance with ARRIVE guidelines and the National Institutes of Health Guide for the Care and Use of Laboratory Animals [29]. Adult male Sprague Dawley rats (250–280 g; 6–8 weeks; Charles River Laboratories, Hollister, CA, USA) were housed individually in standard plastic cages under controlled environmental conditions (22 °C, 40–50% humidity) with a normal 12 h light/dark cycle. Food (Teklad Global 18% Protein Rodent Diet; Envigo, Livermore, CA, USA) and water were provided *ad libitum* for the duration of the experiment.

Animals were randomly assigned to experimental groups (Suppl. Table S1) using a random number generator function in Microsoft Excel. Rats were administered DFP (90% ± 7% pure as determined by ^1^H NMR, Sigma Aldrich, St Louis, MO, USA) at 4 mg/kg, sc, given in the subscapular region as described [30]. DFP was diluted in sterile ice-cold 0.2 M phosphate buffered saline (PBS; 3.6 mM Na_2_HPO_4_, 1.4 mM NaH_2_PO_4_, 150 mM NaCl, pH 7.2). Vehicle (VEH) animals received the same volume (300 µL) of ice-cold PBS. One min later, DFP and VEH animals were administered atropine sulfate (purity 97%, Sigma Aldrich) at 2.0 mg/kg in saline, im, and 2-pralidoxime (2-PAM, purity 97%, Sigma Aldrich) at 25 mg/kg in saline, im. These drugs significantly reduced mortality by blocking the peripheral parasympathomimetic symptoms associated with acute OP intoxication [31].

The severity of seizure behavior was quantified using a 6-point scale as previously described (Fig. 1b) [30, 32]. Seizure behavior was scored at 5 min intervals from 0 to 120 min post-DFP injection and at 20 min intervals from 120 to 240 min post-DFP injection (Fig. 1c). Seizure severity for each animal was quantified as the average seizure severity score, which is the average of the 16 scores collected during the initial 4 h post DFP. Only animals with an average seizure score of 2.5 or higher, which corresponds to electrographic SE [32], were included in further experimentation. At 6 h post exposure, animals were injected sc with 10 mL of 5% w/v dextrose in saline (Baxter, Deerfield, IL, USA) and returned to their home cages. Rats were weighed daily post-exposure and provided moistened rat chow for 3–5 days until they were able to locate and consume standard chow and water independently.

Subsets of animals from each experimental group were euthanized at 1, 3, 7, or 28 days post-exposure via inhalation of 4% isoflurane in medical grade oxygen, and subsequent transcardial perfusion with cold PBS at a flow rate of 15 mL/min using a Masterflex peristaltic pump (Cole Parmer, Vernon Hills, IL, USA). Brains were removed and bisected in the sagittal plane, with one hemisphere dissected on ice to obtain the hippocampus, cortex, and cerebellum, which were snap frozen in liquid nitrogen, and the other hemisphere processed for immunohistochemistry (IHC).

### Enzymatic activity assay and ELISA

Frozen brain tissue was added to 0.1 M Tris-HCl buffer (TBS; 96 mM Tris-HCl, 187 mM NaCl, 50 mL MQ H_2_O, pH 8.5) with 0.1% w/v Triton X-100 (Thermo Fisher) at 0.1 g tissue per 1 mL. Tissue was sonicated using a Virtis ultrasonic probe sonicator at power level 3 with 2–3 s bursts until > 95% of the tissue was homogenized. Sonicated tissue was aliquoted into 1 mL volumes and centrifuged at 21,000 X g for 30 min at 4 °C using an Eppendorf 5417R centrifuge (Eppendorf, Enfield, CT, USA). Total brain supernatant protein and total plasma protein levels were quantified using the Pierce BCA Protein Assay Kit (Thermo Scientific), following the manufacturer’s instructions for microplate assays. Brain supernatant was removed, transferred to microplate wells, and diluted 1:40 to 1:80 with the buffer used for the corresponding ELISAs but without the addition of bovine serum albumin (BSA). ELISA and BCA assays were quantified using a Synergy H1 microplate reader at 450 nm and 562 nm, respectively (BioTek, Winooski, VT, USA).

Plasmin enzymatic activity in blood plasma was measured using a Sensolyte AFC Plasmin Activity Assay kit (Anaspec, Fremont, CA, USA; RRID: SCR_002114) with active plasmin at Vmax determined from a standard curve following the manufacturer’s instructions. The plasmin activity in each sample was normalized to the plasma protein concentration of the same sample, which was determined using the BCA protein assay.

Total protein levels of plasma PAI-1 were measured using the RPAIKT-TOT ELISA kit (Molecular Innovations, Novi, MI, USA), following the manufacturer’s instructions. Total PAI-1 included free PAI-1 and PAI-1 complexed with either tPA or uPA. The total PAI-1 level in each sample was normalized to the plasma protein concentration in the same sample.

Total protein levels of PAI-1, tPA, and uPA in brain homogenates were measured using the RPAIKT-TOT, RTPAKT-TOT, and RUPAKT-TOT ELISA kits (Molecular Innovations), respectively, following the manufacturer’s instructions. The total concentration of each protein of interest (PAI-1, tPA, uPA) for each sample was normalized to total protein concentration in the same sample.

### Immunohistochemistry (IHC)

Brain tissue used for IHC was prepared as previously described [12]. Briefly, brain tissue was sliced into 2 mm sections using a rat brain matrix, fixed in 4% paraformaldehyde for 24 h before being submerged in a 30% sucrose solution for 48 h prior to staining. Then, slides were immunostained in batches that included negative control slides incubated in blocking buffer without primary antibody. Sections were incubated with primary antibody (Suppl. Table S2) in blocking buffer at 4 °C overnight in the dark. The following day, sections were washed four times for 5 min in PBS with Triton X-100 (0.03%) and incubated with the appropriate secondary antibody (Suppl. Table S3) diluted in blocking buffer for 1 h at room temperature in the dark. Antibodies specific for NeuN [33], GFAP [34], IBA1 [35], CD68 [36], CD31 [37], and AQP4 [38] have been previously validated. The specificity of the antibody used to visualize PAI-1 expression in the brain was validated in-house (Suppl. Fig. S1). Slides were mounted in ProLong^tm^ Gold Antifade mounting medium with DAPI (Invitrogen; Waltham, MA) to identify cell nuclei.

### Image acquisition and analysis

Fluorescent images were acquired as previously described [12]. A photographic rat brain atlas [39] was used to confirm anatomical structures, and images of the following brain regions were acquired from Bregma − 2.5 mm to -4.5 mm: (1) dentate gyrus of the hippocampus, (2) thalamus, (3) piriform cortex, 4) CA1 region of the hippocampus, (5) CA3 region of the hippocampus, and (6) amygdala. Immunoreactivity was assessed with respect to the percentage of cells in the field of view (identified by DAPI staining) that were immunopositive for the biomarker of interest using the Multi Wavelength Cell Sorting Journal within the Custom Module Editor image analysis software (MetaXpress High-Content Image Acquisition and Analysis software, version 6.1, Molecular Devices). The custom modules used for these analyses can be found in Suppl. Method 1. Representative images of all experimental groups were acquired from the same slides used for high content quantification using a Leica SP8 STED microscope equipped with a 63x objective available through the Advanced Imaging Facility Core at the University of California, Davis. These images were edited using FIJI software version 2.1.0/1.53c.

### Transmission electron microscopy (TEM)

A separate animal cohort was used to collect samples for EM because of the differences in sample preparation needed for EM imaging. Sprague Dawley rats were exposed to DFP (*n* = 1) or VEH (*n* = 1), as described earlier (Fig. 1a). Their brains were collected for TEM at 1 DPE. Samples were perfused as described above. Coronal brain Sect. (2 mm thick) of the hippocampus and piriform cortex at -3.0 mm Bregma were post-fixed in 2.5% v/v glutaraldehyde (Ted Pella, Redding CA, USA), 2% w/v paraformaldehyde (PFA; Ted Pella) in 0.1 M PBS (26 mM NaH_2_PO_4_, 77 mM Na_2_HPO_4_, 400 mL MilliQ H_2_O, pH 7.3) provided by the Biological Electron Microscopy Facility Core at the University of California, Davis. After fixation for 96 h at 4 °C, tissues were rinsed twice in 0.1 M PBS for a total of 30 min and then placed in 1% w/v osmium tetroxide (Electron Microscopy Sciences, Hatfield, PA, USA) in 0.1 M PBS for 1 h. Tissues were rinsed twice for 15 min each in 0.1 M PBS. The samples were next dehydrated in a series of graded ethanol (50%, 75%, 95% for at least 30 min each and 100% twice for 20 min). Tissues were washed twice in propylene oxide (Electron Microscopy Sciences) for 15 min and then pre-infiltrated in half resin (composed of 450 mL dodecenyl succinic anhydride, 250 mL araldite 6005, 82.5 mL Epon 812, 12.5 mL dibutyl phthalate, and 450 uL benzyl dimethylamine; Electron Microscopy Sciences) and half propylene oxide overnight. The following day, tissues were infiltrated in 100% resin for 5 h. The tissues were embedded with fresh resin and polymerized at 60 °C overnight. The embedded tissues were sectioned using a Leica EM UC6 ultramicrotome (Leica Biosystems Inc., Buffalo Grove, IL, USA) at a thickness of 90 nm and collected on copper mesh grids. Sections were stained with 4% w/v aqueous uranyl acetate (Ted Pella, Redding, CA, USA) for 20 min and for 2 min in 0.2% w/v lead citrate (Eastman, Kingsport, TN, USA) in 0.1 N NaOH. Representative images were acquired at the Biological Electron Microscopy Facility Core (https://bioem.ucdavis.edu) at the University of California, Davis, using a FEI Talos L120C transmission electron microscope at 80kv and Thermo Scientific Ceta 16MP camera (Thermo Scientific, Waltham, MA, USA).

### RT- qPCR

Brain tissue from the cortical and hippocampal regions from a small subset of 1 DPE VEH (*n* = 2) and 1, 3, 7, or 28 DPE DFP (*n* = 2–3 per time point) animals was taken for analysis by RT-qPCR. Samples were homogenized in mortar and pestles with liquid N_2_. RNA was subsequently extracted with Trizol (ThermoFisher Scientific, Catalog # 15596026) according to the manufacturer’s protocol. RNA purity and concentration were assessed using a Nanodrop Spectrophotometer ND-1000 (ThermoFisher Scientific). The 260 nm/280 nm absorption ratios for the samples ranged from 1.90 to 2.00. Subsequently, a cDNA library was made with a High-Capacity cDNA Reverse Transcription Kit (ThermoFisher Scientific, Catalog# 4374967), with 2 µg of total RNA per 20 uL reaction. Reactions were carried out in a PTC-200 Peltier Thermal Cycler; the conditions were as follows: 25 °C for 10 min, 37 °C for 120 min, 85 °C for 5 min, with final storage at 4 °C. RT-qPCR reactions were conducted in triplicate in an Applied Biosystems Viia7 Real-Time PCR System using Maxima SYBR Green/ROX qPCR Master Mix (ThermoFisher Scientific, Catalog #K022) and with the recommended three-step cycling protocol. CT values were normalized to the geometric mean of three housekeeping genes: β*-actin*, *hmbs*, and *ywhaz*. RT-qPCR primer sequences are provided in Suppl. Table S4.

### Statistics

Plasmin Activity Assay: Mixed effect models, including animal-specific random effects, were used to assess differences between DFP and VEH groups and time post-exposure (1, 3, 7, or 28 days post-exposure). Data were transformed logarithmically to fit model assumptions. Akaike Information Criterion (AIC) was used to find the best model for each outcome [40]. Benjamini-Hochberg False Discovery Rate (FDR) was used to determine comparisons that remained significant after accounting for multiple testing. Results are presented as the geometric mean ratio (GMR), including the 95% confidence interval, between DFP and VEH. When the confidence interval for the GMR includes 1, no statistically significant difference exists between compared groups. R Studio (version 3.6.0, R Core Team (2019), Vienna, Austria) was used for all analyses and to output graphics.

ELISA: Mixed effect models, including animal-specific random effects, were used to assess differences between DFP and VEH groups by brain region (cerebellum, cortex, hippocampus) and time post-exposure (1, 3, 7, or 28 days post-exposure). All outcomes of interest (PAI-1, tPA, and uPA) were transformed, checked for best model fit, and run through post-hoc analysis using the same methods described for the plasmin activity. SAS software (version 9.4, SAS Institute, Inc., Cary, NC, USA) was used for all analyses, and RStudio (version 3.6.0, R Core Team (2019), Vienna, Austria) was used to output graphics. Sample sizes were determined a priori using a power analysis software (G*Power version 3.1) [41]. The two-tailed t-test of the differences between two independent means used an effect size of 2, an alpha of 0.05, a power level of 0.8, and an allocation ratio of 2. Specific ELISA values and GMR values are given in Suppl. Tables S5-S10.

Quantitative IHC: Primary outcomes were pairwise co-localizations of PAI-1 and GFAP, PAI-1 and IBA1, PAI-1 and CD68, and PAI-1 and NeuN. Mixed effects models, including animal-specific random effects, were fit to assess differences between exposure groups. Primary factors of interest included exposure (DFP, VEH), region (amygdala, CA1, CA3, dentate gyrus, piriform cortex, thalamus), and time post exposure (1, 3, 7, or 28 days post-exposure). Interactions between the factors (exposure, region, and time point) were considered to choose the best model using AIC. The outcome was transformed using the natural logarithm after shifting all values by a small number to better meet the assumptions of the model. Animals or regions with no co-localization were shifted by minute amounts (0.1 for PAI-1/IBA-1 and PAI-1/CD68 colocalization, 0.5 for PAI-1/NeuN, and 1 for PAI-1/GFAP colocalization) to bring numbers above 0 to allow for natural logarithmic transformation. Contrasts for group differences were constructed and tested using a Wald test. Significance was assessed and presented using the same post-hoc analysis and graphical methods described for the plasmin activity. All analyses were performed using SAS software (version 9.4) with alpha set to 0.05. Results remained significant after implementation of the FDR post-hoc analysis unless otherwise stated.

RT-qPCR: All CT values were normalized to the geometric mean of three housekeeping genes, *β-actin*, *hmbs*, and *ywhaz*, as recommended by Vandesompele, et al. [42]. Fold changes were calculated by the ∆∆Ct method; relative mRNA levels were generated by normalizing control Sprague Dawley rat brains. Primers were purchased from BioRad as part of their PrimePCR plate and sequences are provided in Suppl. Table S11. Statistical analysis comparing the hippocampus or cortex of the DFP treatment group at various time points to the hippocampus or cortex of vehicle treated animals was performed in Prism using Welch’s unpaired t-test.

## Results

### Acute DFP intoxication activated the peripheral PAS

**Fig. 2:**
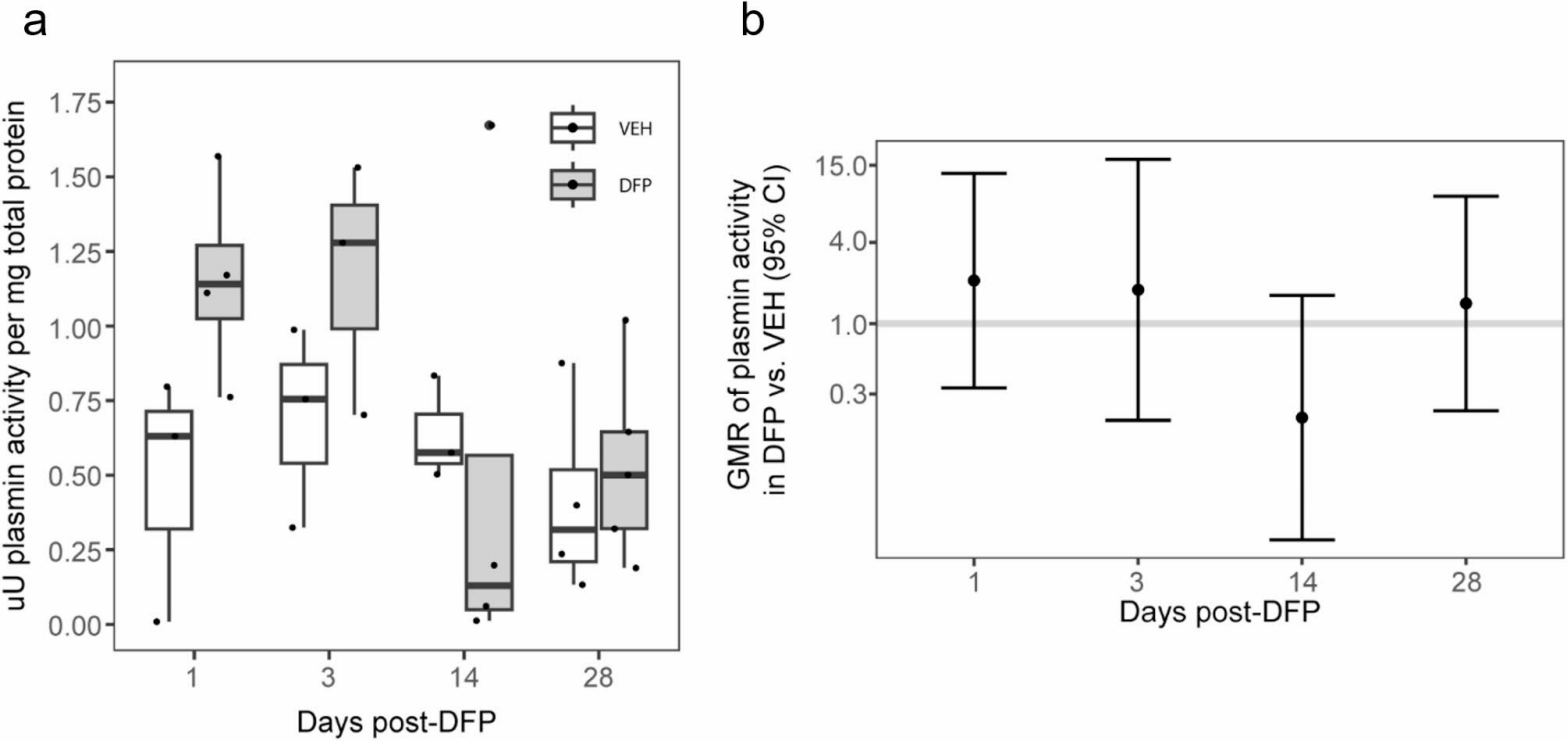
Acute DFP intoxication altered plasmin activity in plasma (**a**) Active plasmin enzymatic content normalized to total plasma protein in VEH (white, *n* = 3–5) and DFP (grey, *n* = 3–5) animals at varying DPE. Data are presented as box plots in which dots represent individual animals; the box plot bounds, the interquartile range (IQR); the horizontal line in each box, the median; and the whiskers extend to the last observation within 1.5 x the IQR. (**b**) Geometric mean ratios (GMR, dot) and 95% CI (bar) of normalized plasmin activities in plasma from DFP vs. VEH animals at specific DPE. If the 95% CI crosses the horizontal line at 1.0, there was no significant difference between DFP and VEH animals

**Fig. 3:**
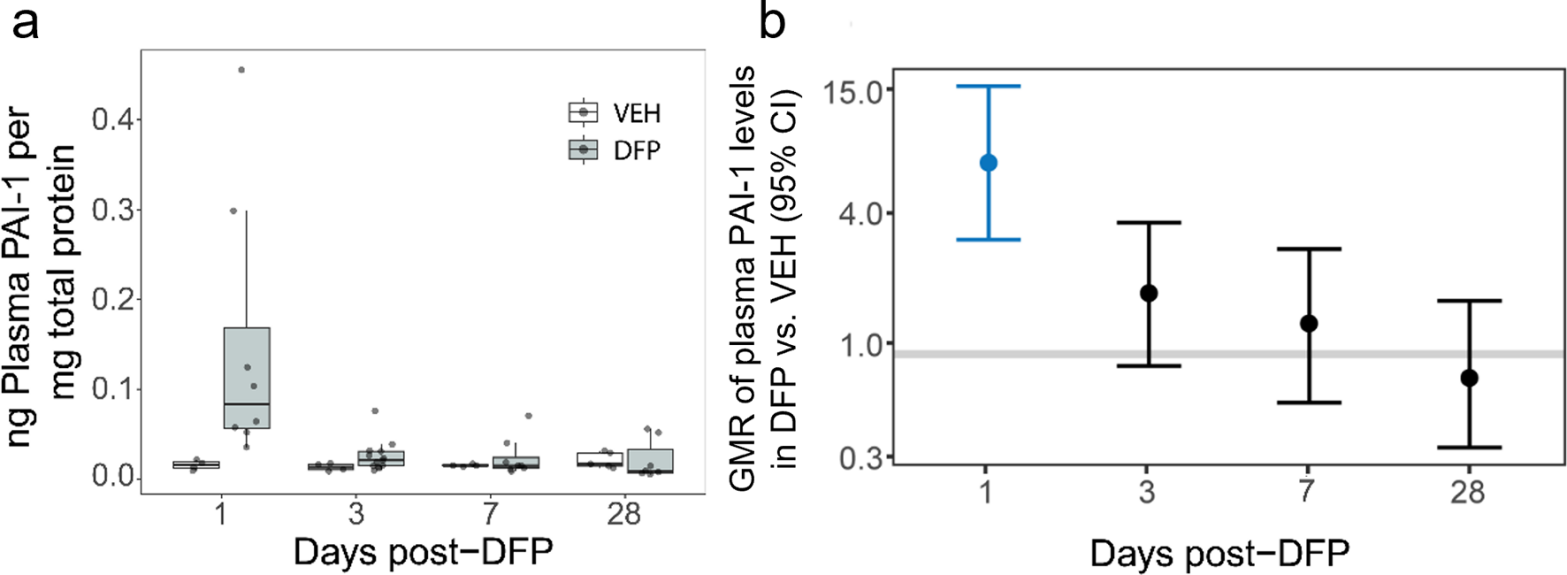
Temporal profile of DFP effects on plasma PAI-1 levels (**a**) Plasma PAI-1 levels were quantified by ELISA and normalized to total plasma protein. Data are presented as box plots of VEH (white, *n* = 4–5) and DFP (grey, *n* = 7–13) animals. Dots represent an individual animal; box plot bounds, the interquartile range (IQR); the horizontal line within the box, the median; and the whiskers extend to the last observation within 1.5 x the IQR. (**b**) Geometric mean ratio (GMR, dot) and 95% CI (bar) of the total plasma PAI-1 in DFP vs. VEH animals at 1, 3, 7, and 28 DPE. A GMR with a 95% CI that crosses the horizontal line at 1.0 indicates no significant difference. If the 95% CI lies entirely above or below the 1.0 line, there was a significant increase or decrease, respectively, in DFP animals relative to VEH. Blue CIs indicate significant differences between DFP and VEH groups after FDR correction (*p* < 0.05)

Evidence of increased incidence of deep vein thromboses in patients who survived acute OP intoxication [19] suggests that this chemical exposure causes aberrant regulation of the PAS. To determine whether acute DFP intoxication affects the PAS, plasmin enzymatic activity was measured in plasma from VEH and DFP animals at 1, 3, 7, and 28 days post-exposure (DPE). Acute DFP intoxication caused an apparent increase in plasmin activity at 1 and 3 DPE but not at 7 or 28 DPE (Fig. 2); however, group differences were not statistically significant. When normalized to plasma protein content, plasmin activity in the plasma of DFP-intoxicated rats was highly variable, ranging from below basal levels measured in VEH animals to a more than 2-fold increase relative to VEH. This high variability in normalized plasmin activity suggested the possibility that expression of the principal inhibitor of plasminogen activation, PAI-1, was dysregulated following acute OP intoxication. ELISA measurements of total plasma PAI-1 protein, normalized to plasma protein, demonstrated a significant, 10-fold increase in PAI-1 levels in DFP animals compared to VEH at 1 DPE (Fig. 3). In addition, there was a 2-fold elevation at 3 DPE before PAI-1 protein returned to basal levels at 7 and 28 DPE.

### Acute DFP intoxication altered brain levels of plasminogen activators and PAI-1

**Fig. 4:**
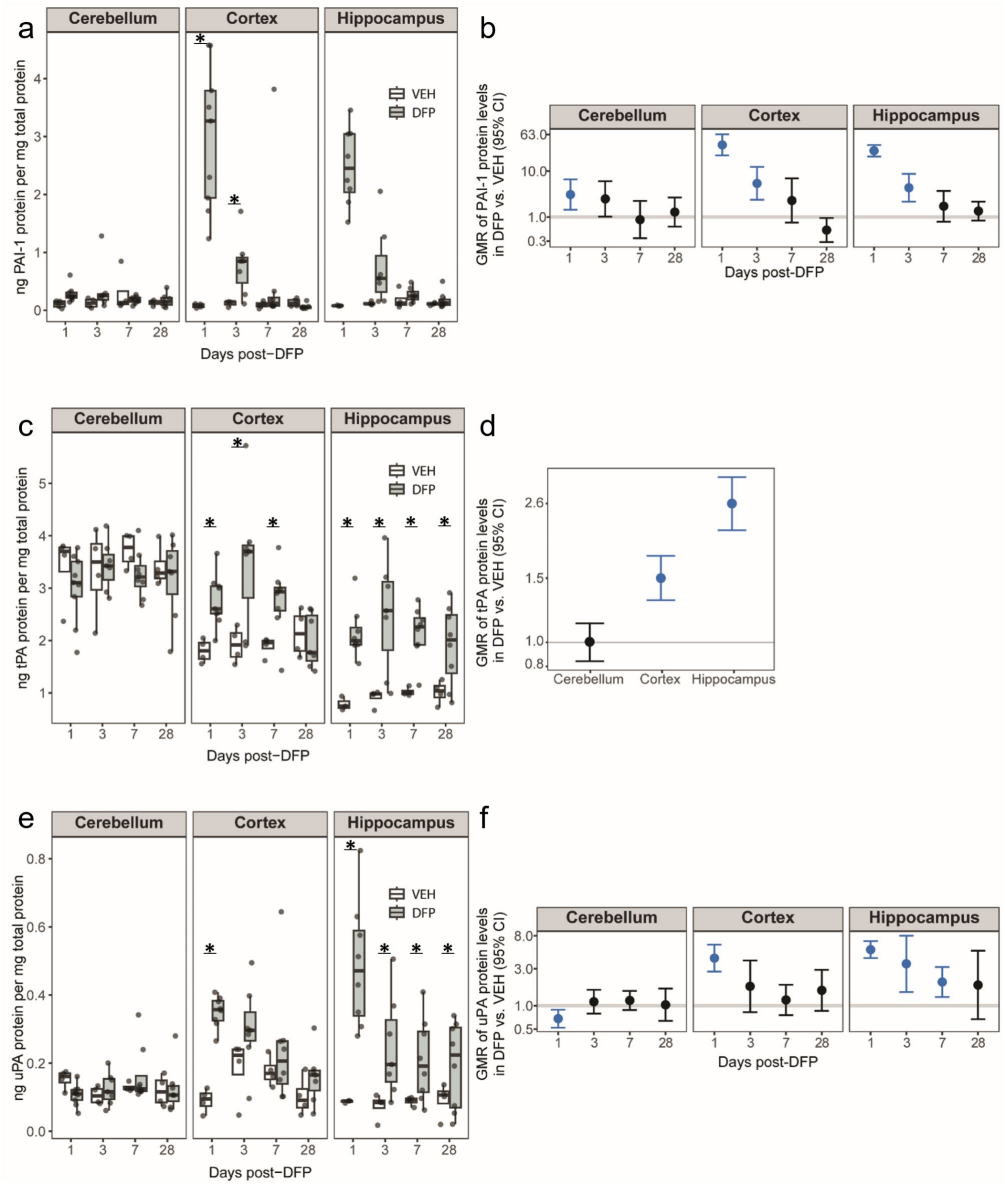
Acute DFP intoxication perturbed multiple components of the plasminogen activating system in a time- and region-dependent manner Protein levels of plasminogen activator inhibitor type 1 (PAI-1), tissue plasminogen activator (tPA), and urokinase plasminogen activator (uPA) in various brain regions were quantified by ELISA and normalized to total protein content. (**a**, **b**) PAI-1, (**c, d**) tPA, and (**e**, **f**) uPA protein levels in the cerebellum, cortex and hippocampus of VEH (white, *n* = 3–4) and DFP (grey, *n* = 7–9) animals at varying DPE. (**a**, **c**, **e**) Data are presented as box plots where dots represent individual animals; box plot bounds, the interquartile range (IQR); the horizontal line in the box, the median; and the whiskers extend to the last observation within 1.5x the IQR. * Indicates a significant difference in protein level between treatment groups at the same time point, as determined by mixed-effects analysis and FDR correction (*p* < 0.05). (**b**, **d**, **f**) Geometric mean ratios (GMR, dot) and 95% CI (bar) of normalized PAI-I, tPA or uPA levels in DFP vs. VEH brains at specific DPE. If the 95% CI crosses the horizontal line at 1.0 there was no significant difference between DFP and VEH animals. Blue CIs indicate a statistically significant difference between DFP and VEH animals. Note that in panel d, differences in tPA levels between groups did not vary significantly by DPE, so an overall estimate of the group differences by brain region is presented

To determine whether components of the PAS were also perturbed in the brain following DFP-induced SE, levels of latent, free and complexed PAI-1, tPA, and uPA were measured by ELISA in the cerebellum, cortex, and hippocampus of DFP and VEH animals. Consistent with the shifts in PAI-1 seen in blood plasma, total PAI-1 brain protein levels, normalized to total protein content, were significantly increased (*p* < 0.05) at 1 DPE in all three brain regions, with increases ranging from 3- to 37-fold (Fig. 4a-b). At 3 DPE, total PAI-1 levels in the cortex and hippocampus remained elevated relative to VEH (*p* < 0.05) but were decreased relative to 1 DPE, whereas PAI-1 levels in the cerebellum returned to VEH level. By 7 DPE, PAI-1 levels in the cortex and hippocampus had also returned to VEH levels and remained so at 28 DPE.

Regional differences in tPA levels were observed in VEH animals, with the highest tPA levels observed in the cerebellum and lowest levels in the hippocampus. Acute DFP intoxication altered tPA levels in a region-dependent manner. Cortical tPA levels were significantly elevated by DFP at 1, 3, and 7 DPE (*p* < 0.05) before returning to control levels by 28 DPE (Fig. 4c-d). In contrast, hippocampal tPA protein levels remained elevated at all time points post-DFP-intoxication. In the cerebellum, no statistical differences were found in tPA levels between DFP and VEH animals at any time post-exposure.

Brain protein levels of total uPA were 10-fold lower than total tPA in all brain regions (Fig. 4c vs. 4e). Acute DFP intoxication significantly depressed uPA levels in the cerebellum at 1 DPE but levels returned to control by 3 DPE. In contrast, DFP increased uPA levels in the cortex and hippocampus (*p* < 0.05). In the cortex, DFP effects were transient, evident as a 4-fold increase at 1 DPE with a return to control by 3 DPE. In the hippocampus, uPA levels increased 5-fold at 1 DPE and remained significantly elevated through 7 DPE (Fig. 4e-f).

### Spatiotemporal changes in PAI-1 expression following acute DFP intoxication

**Fig. 5:**
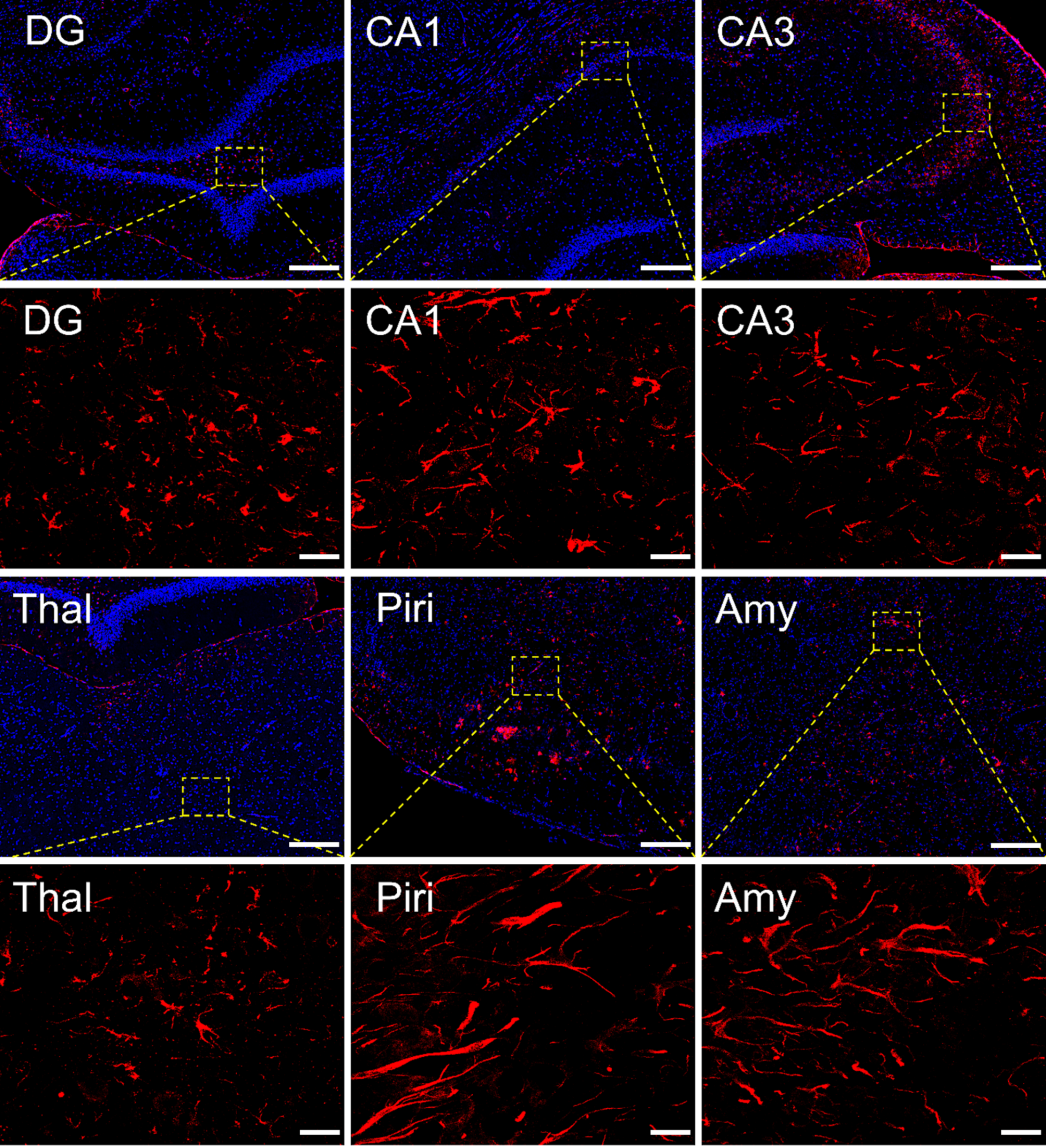
Acute DFP intoxication induced PAI-1 expression in multiple brain regions at 28 DPE Representative photomicrographs of PAI-1 (red) immunoreactivity to identify PAI-1 protein in the dentate gyrus (DG), CA1 and CA3 regions of the hippocampus, thalamus (Thal), piriform cortex (Piri), and amygdala (Amy) at 28 DPE. Sections were counterstained with DAPI (blue) to identify cell nuclei. Boxed areas are shown at higher magnification in the image below. Low magnification bars = 200 μm, high magnification bars = 20 μm

To determine whether the elevated PAI-1 expression localized to brain regions previously reported to exhibit extensive neurodegeneration, microgliosis, and reactive astrogliosis following acute DFP intoxication [12, 30], PAI-1 expression in the hippocampus (dentate gyrus, CA1, and CA3 regions), thalamus, piriform cortex, and amygdala was analyzed by IHC at 1, 3, 7 and 28 DPE. Basal levels of PAI-1 immunostaining were very low in VEH animals (Suppl. Fig. S2) at all time points. In DFP animals, at 1 and 3 DPE, weak PAI-1 positive immunoreactivity was observed in the dentate gyrus and CA1 subregions of the hippocampus, the thalamus, and the amygdala, but no PAI-1 immunostaining was observed in the CA3 subregion of the hippocampus or piriform cortex. At 7 DPE, PAI-1 immunoreactivity was found in all brain regions examined except the thalamus, which contained scattered immunoreactivity, adjacent to blood vessels. At 28 DPE, the strongest PAI-1 immunostaining was found (Fig. 5) in the hilus of the dentate gyrus, concentrated towards the tip, the CA1, CA3, and thalamic regions. Interestingly, positive PAI-1 immunostaining observed in the piriform cortex and amygdala region seems morphologically distinct from the PAI-1 staining observed in other regions. This likely reflects known differences in the vasculature [43–45], as well as the function and morphology of astrocytes [46–48] in these brain regions. Additionally, the amygdala and piriform cortex exhibit the most extensive neuropathology following acute DFP intoxication [30] suggesting more robust astrogliosis. Additional images of PAI-1 immunostaining in all brain regions at all time points are provided in Suppl. Figures 3, 4.

**Fig. 6:**
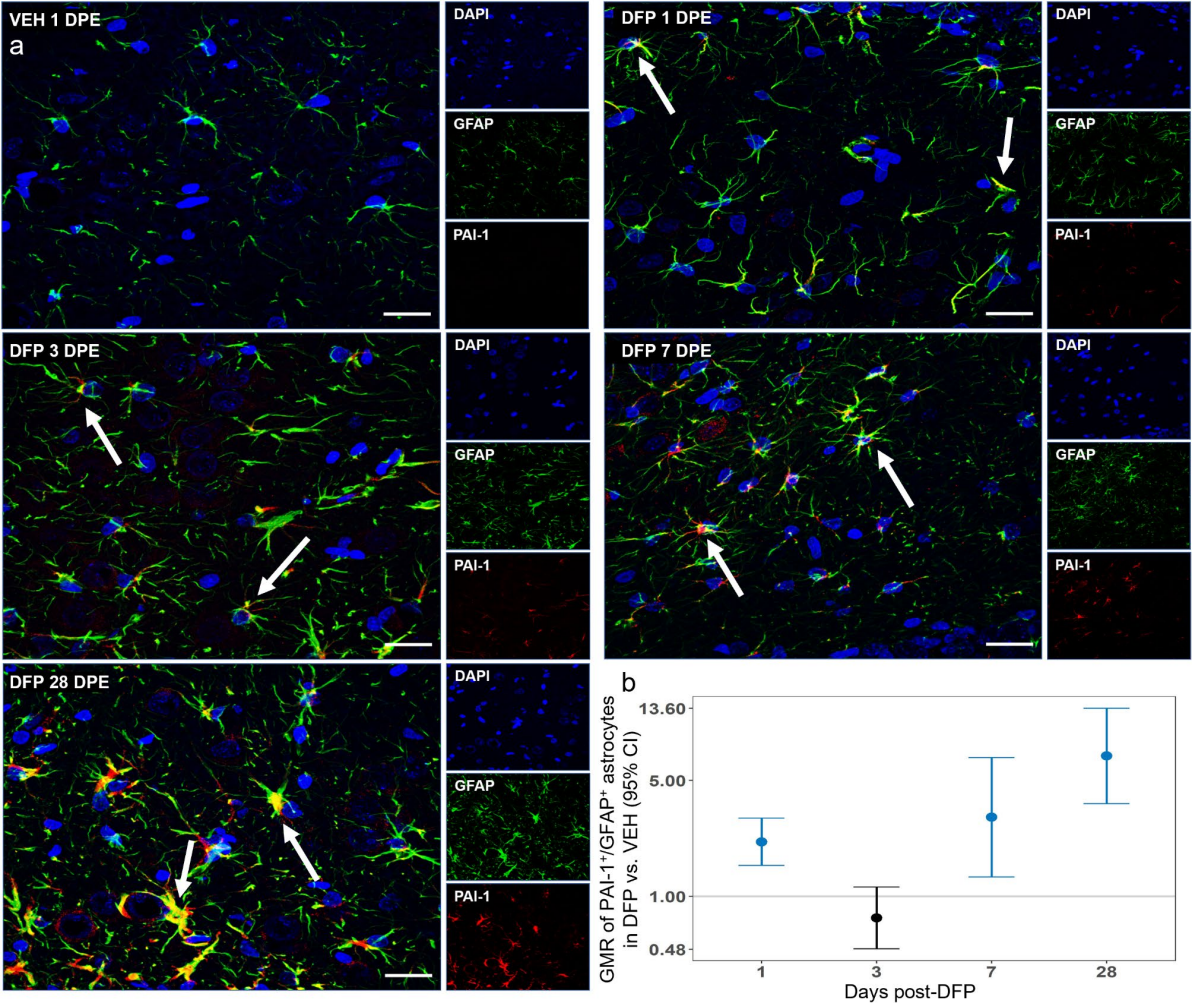
Acute DFP intoxication induced PAI-1 expression in astrocytic sub-populations (**a**) Representative confocal photomicrographs of PAI-1 (red) and GFAP (green) immunoreactivity in the dentate gyrus of a VEH animal at 1 DPE and DFP animals at 1, 3, 7 and 28 DPE. Sections were counterstained with DAPI (blue) to identify cell nuclei. Bar = 20 μm. Arrows indicate PAI-1 positive staining in astrocytic cell bodies and processes. (**b**) Geometric mean ratio (GMR, dot) and 95% CI (bar) of PAI-1 and GFAP colocalization in DFP (*n* = 10–12) vs. VEH (*n* = 10–12) animals, as determined by high content image analysis. Differences between groups did not vary by brain region; therefore, overall differences between groups are presented as a function of DPE. If the 95% CI falls entirely above or below the horizontal line at 1.0, there was a significant increase or decrease, respectively, between DFP and VEH animals. CIs in blue indicate significant difference between DFP and VEH animals at *p* < 0.05 after FDR correction

PAI-1 immunostaining was predominantly found in distal cellular processes, particularly adjacent to blood vessels, with some additional expression in cellular bodies in the hilus of the dentate gyrus. To determine which cell types in the brain were immunoreactive for PAI-1, brain sections were co-labeled for PAI-1 and cell type-specific biomarkers: NeuN to identify neurons [49]; IBA-1, microglia [50]; CD68, phagocytic cells [51]; and GFAP, astrocytes [52]. Laser confocal microscopy was used to acquire images in multiple z-planes to identify colocalization of PAI-1 with cell type-specific biomarkers in stacked z-planes. Analysis of these confocal images indicated PAI-1 immunoreactivity was physically adjacent to but not colocalized in NeuN^+^ neurons (Suppl. Figure 6). The same was true for IBA-1 immunopositive microglia and CD68 immunopositive microglia (Suppl. Figure 7). In contrast, PAI-1 co-localized with GFAP immunoreactivity (Fig. 6a). PAI-1^+^/GFAP^+^ co-labeling was identified within subpopulations of astrocytes in all brain regions examined, consistent with previous observations of sustained astrogliosis in these brain regions following acute DFP intoxication [12, 13, 17, 30]. After determining that PAI-1 was colocalized with GFAP^+^ astrocytes via confocal microscopy, slides were analyzed with a high content imaging system to quantify the level of colocalization. PAI-1 expression in GFAP^+^ astrocytes was significantly increased at 1 DPE, subsided at 3 DPE, but then progressively increased at 7 and 28 DPE (Fig. 6b). Group differences in PAI-1^+^/ GFAP^+^ colocalization did not vary between brain regions.

**Fig. 7:**
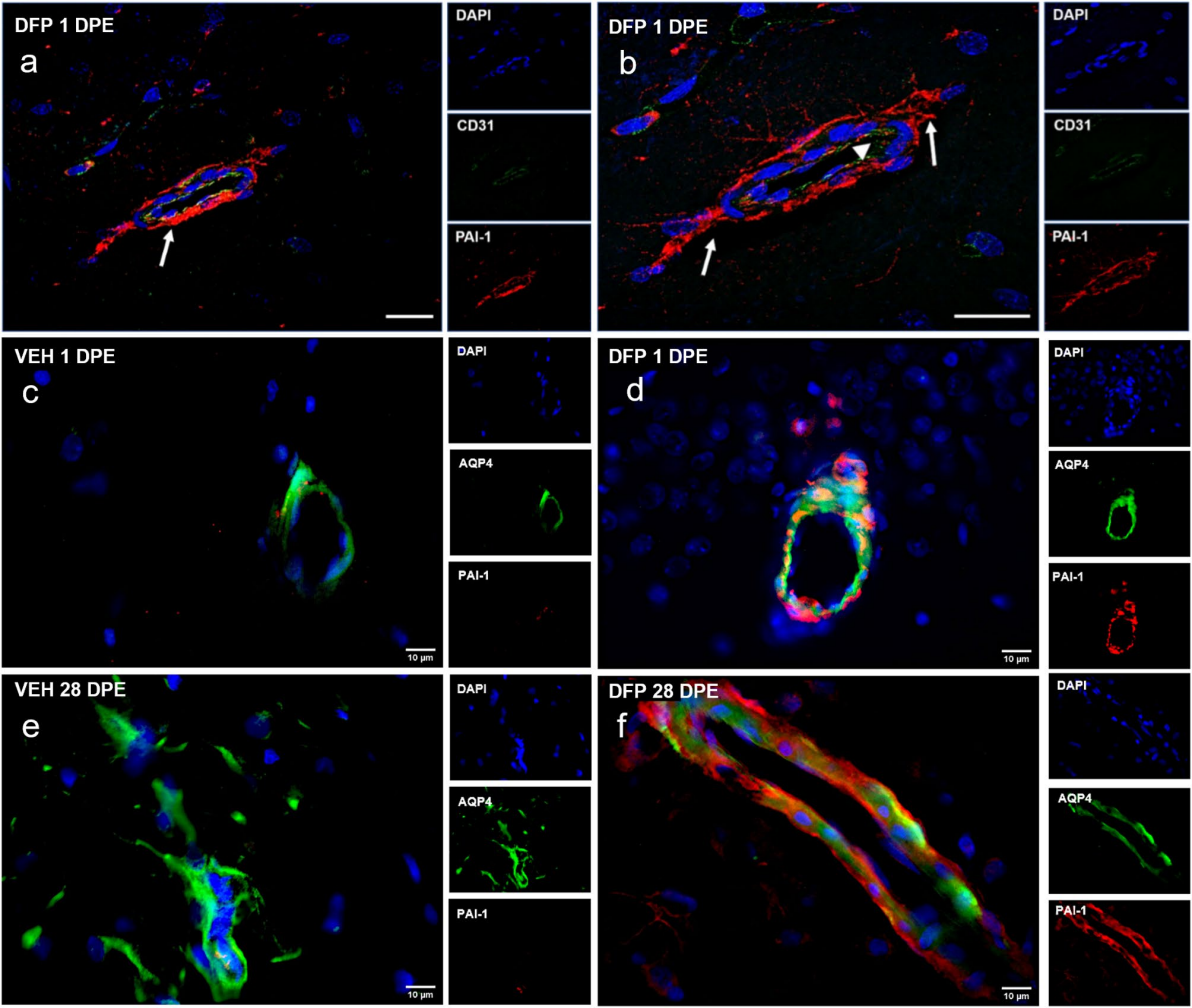
PAI-1 expression was localized to specific cell types in the brain of DFP-intoxicated animals (**a**) Representative photomicrographs of PAI-1 (red) and CD31 (green) immunoreactivity in the hippocampus of a DFP-intoxicated animal at 1 DPE. Sections were counterstained with DAPI (blue) to identify cell nuclei. Bar = 20 μm. (**b**) A higher magnification image of (**a**). Arrows indicate PAI-1 immunoreactivity is adjacent to but not present within arteriolar blood vessels (arrowhead). (**c-f**) Representative photomicrographs of PAI-1 (red) immunoreactivity and aquaporin 4 (AQP4, green) immunoreactivity. AQP4 identifies astrocytic end feet on the abluminal surface of vessels in a VEH **(c**,** e)** and DFP (**d**,** f**) animal at 1 and 28 DPE

Confocal microscopy performed on brain sections of DFP-intoxicated rats confirmed PAI-1 immunostaining in extended processes of GFAP^+^ astrocytes and in some distal astrocytic processes surrounding and contacting arteriolar and capillary blood vessels. Immunoreactivity for PAI-1 was specifically noted in distal astrocytic processes and their astrocytic end feet in close physical apposition to arteriolar endothelial cells expressing the endothelial cell marker, CD31 [53], but not within the endothelial cells themselves (Fig. 7a-b). In contrast, immunostaining for aquaporin-4 (AQP4), a protein expressed by astrocytic end feet on the abluminal surface of vessels [54], was colocalized with PAI-1 immunoreactivity (Fig. 7c-d), verifying that PAI-1 was expressed within the astrocytic end feet.

### Acute neuronal injury to the arteriolar neurovascular unit following DFP intoxication

**Fig. 8:**
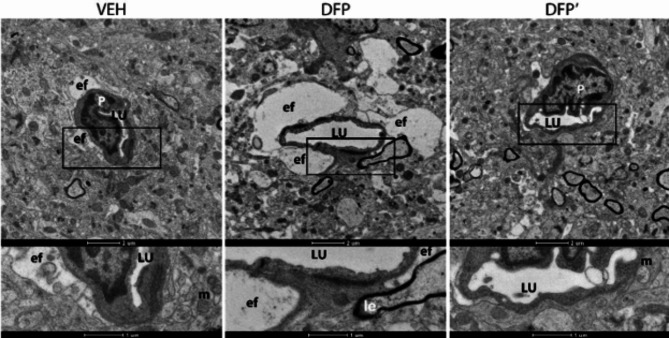
DFP induced cellular damage in the hippocampus Transmission electron micrographs of the dentate gyrus in VEH or DFP at 1 DPE (DFP’: same animal, different region of hippocampus). Intra-myelinic edema (Ie; middle lower panel) was noted in the DFP brain compared to a time matched VEH control. Astrocytic mitochondria (m) in the DFP brain exhibited loss of cristae resolution not seen with the VEH control. Astrocytes in the DFP animal also presented swollen astrocytic end feet and edema. There were no signs of endothelial damage or loss of tight junctions in DFP or VEH tissue. LU, vascular lumen; P, pericyte; ef, end feet

To determine whether DFP-induced expression of PAI-1 on arterioles and capillaries reflected damage to the neurovascular unit, hippocampal tissue from DFP-intoxicated animals at 1 DPE was examined by transmission electron microscopy (TEM). TEM images demonstrated evidence of astrocytes with swollen end feet, mitochondrial edema, and loss of cristae resolution that was not observed in hippocampal tissue from VEH controls at 1 DPE (Fig. 8). Acute DFP intoxication also induced signs of intramyelinic edema in the hippocampus. There was no observable evidence of endothelial damage or loss of tight junctions, which form a physical barrier as part of the NVU.

### TGF-β transcription was increased in the DFP-intoxicated brain

**Fig. 9:**
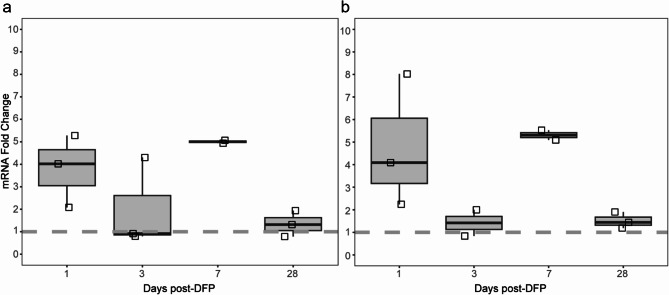
Acute DFP intoxication upregulated TGF-β transcripts in the brain as determined by qPCR TGF-β mRNA was quantified in the hippocampus (**a**) and cortex (**b**) of DFP animals at 1, 3, 7 and 28 DPE (*n* = 2–3) and normalized to TGF-β levels in the corresponding brain regions of VEH animals at 1 DPE (*n* = 2). Data are presented as box and whisker plots in which squares represent individual animals; the ends of the whiskers, the minimum and maximum values; and the horizontal line in the box, the median. The dashed line at y = 1 represents the baseline value from 1 DPE VEH animals

DFP-induced neuroinflammation has previously been reported to produce prominent regional brain astrogliosis that progressively increases over time [13, 14, 16, 30]. TGF-β is considered a primary driver of reactive gliosis [55], and its intracellular active form rapidly activates the SERPIN 1 promoter encoding PAI-1 [56, 57]. Because we observed increasing populations of GFAP^+^/PAI-1^+^ astrocytes post-DFP, TGF-β transcript levels were quantified in the hippocampus (Fig. 9a) and cortex (Fig. 9b) at varying DPE. Acute DFP intoxication significantly increased TGF-β mRNA in both brain regions at 1 and 7 DPE, measured as a fold change from its respective control VEH rat brain region (hippocampus or cortex.

## Discussion

To our knowledge, this is the first report demonstrating that acute OP intoxication causes abnormalities of the PAS. Immediately following acute DFP intoxication, plasma levels of enzymatically active plasmin demonstrated marked variability when compared with VEH animals. This variation in plasmin activities can be attributed, at least in part, to 4- to 15-fold increases in plasma PAI-1 levels. PAI-1 is the principal inhibitor of vascular plasmin activation, and elevated plasma levels of PAI-1 are clinically associated with inhibition of fibrinolysis and increased risk of thrombus formation [58–60]. Clinically, deep vein and pulmonary thrombosis have been reported as statistically significant complications following acute OP intoxication [19, 61].

The marked elevation of plasma PAI-1 levels at 1 DPE was mirrored by an acute elevation of pro-inflammatory PAI-1 levels in the brain that varied in a time- and region-dependent manner. Why the effect of DFP varies regionally is not known but likely reflects the fact that the principal molecular target of DFP, acetylcholinesterase, is not evenly distributed throughout the brain. Plasminogen activation in the central nervous system (CNS) interstitial space is tightly regulated by the irreversible inactivation of tPA and uPA when PAI-1 binds to their active sites [62]. Latent and active forms of free PAI-1 are short-lived, with half-lives measured in hours [59, 63], such that by 1 DPE much of total brain PAI-1 would be expected to be complexed with tPA or uPA. Accordingly, elevations of total tPA and uPA protein levels in the hippocampus and cortex temporally mirror elevations of PAI-1 at 1 and 3 DPE. Basal brain levels of tPA were 10-fold higher than uPA in VEH animals, consistent with other reports [64]. tPA in the hippocampus and cortex increased 2- to 3-fold at 1 and 3 DPE, respectively, and remained elevated in the hippocampal region throughout the 28-day period.

The acute increases in PAI-1 at 1–3 days post-DFP parallel the 3-fold increases seen in hippocampus after pilocarpine-induced SE in mice at the same timepoints [27], although increases in tPA levels after pilocarpine were delayed relative to that seen after DFP. In contrast to the current results, following pilocarpine-induced SE, hippocampal tPA levels were decreased at 3 and 24 h and did not increase until 7 days. Our findings in the DFP model are congruent with reports that brain tPA levels are elevated in various neuroinflammatory diseases, including multiple sclerosis and encephalitis [65], excitotoxic injury [66–68], ischemic brain injury [69], and traumatic brain injury [70]. uPA levels significantly increased 5-fold in the cortex and in the hippocampus at 1 and 3 DPE. Levels of uPA and tPA remained significantly elevated in the hippocampus throughout the entire 28-day period. Unexpectedly, uPA levels dropped below comparable VEH levels in the cerebellum at 1 DPE before returning to basal levels by 3 DPE. The uPA observations are consistent with reports that uPA has low basal expression in selective neurons and astrocytes in normal adult brain but is upregulated in some neuroinflammatory disorders, as reported for multiple sclerosis and epilepsy [62, 71].

The increased PAI-1 expression in the piriform cortex, CA1 and CA3 hippocampal regions, amygdala, and thalamus was predominantly localized to astrocytes with negligible levels associated with neurons, microglia or endothelial cells. Over the 28 days following DFP exposure, PAI-1 immunostaining became increasingly restricted to populations of GFAP immunopositive astrocytes within gliotic regions of the dentate gyrus of the hippocampus and somatosensory cortex [13, 14, 17, 72–76]. This aberrant PAI-1 expression was observed in distal astrocytic processes in the hippocampus and in astrocytic end feet in contact with arteriolar endothelial cells. Laser confocal microscopy confirmed that PAI-1 immunoreactivity co-localized with aquaporin-4, which is a biomarker of astrocytic endfeet. Electron microscopic analyses of the hippocampus following acute DFP intoxication revealed edematous astrocytic end feet with mitochondrial swelling consistent with damage to the arteriolar neurovascular unit.

TGF-β is a key driver of astrocyte reactivity [55] and glial scar formation; TGF-β also upregulates GFAP and SERPIN 1 gene expression [55, 77–79]. Our group recently reported that acute DFP intoxication increases BBB permeability as evidenced by the presence of albumin in the brain parenchyma in the piriform cortex, amygdala, thalamus, hippocampus, and cerebral cortex [80]. Uptake of albumin by astrocytes has been shown to activate TGF-β/ALK5 intracellular signaling pathways [81, 82]. Here, we demonstrated that acute DFP intoxication significantly increased TGF-β transcript levels in the hippocampus and cortex at 1 and 7 DPE. Elevated expression of TGF-β following DFP intoxication is consistent with studies reporting astrocytic reactivity associated with TGF-β activation and concomitant astrocytic PAI-1 expression [55, 56]. Determining the causal relationships between the PAS, TGF-β, neuroinflammation and BBB permeability following acute OP intoxication is the focus of future investigations.

Whether the net effects of DFP-induced changes in the PAS in the brain are pathologically significant are unclear. Since we were unable to measure plasmin activity in the brain, and the effects of acute DFP intoxication on plasmin activity in the plasma were variable but not statistically significant, we cannot draw any conclusions on whether acute OP intoxication alters this functional aspect of the PAS. However, PAI-1, uPA, and tPA can influence neuroinflammation and BBB permeability via plasmin-independent mechanisms [20]. PAI-1, an acute phase pro-inflammatory reactant, enables neutrophil infiltration of microvascular endothelial cells following reperfusion-injury to promote vascular leakage and activate microglia [25, 26]. Taken together, these data support the hypothesis that elevations in brain PAI-1, tPA, and uPA following acute OP intoxication could help maintain the protracted neuroinflammation and BBB leakage described in the literature, with or without modulation to plasmin enzymatic activity [30, 80].

In summary, our data provide evidence that acute OP intoxication perturbs the PAS in the brain in a time- and region-dependent manner. These observations suggest that the PAS warrants further investigation as a potential therapeutic target for mitigating the chronic neurotoxic effects of acute OP intoxication. Further, these findings identify PAI-1 as a potential biomarker of damage to the neurovascular unit at the arteriolar level.

## Electronic supplementary material

Below is the link to the electronic supplementary material.

Supplementary Material 1

## Data Availability

Data and materials will be made available upon reasonable request to the corresponding author.
