# Supplementary material for: Spatiotemporal perturbations of the plasminogen activation system in a rat model of acute organophosphate intoxication
Source: Acta Neuropathol Commun. 2025 Mar 18;13:62. doi: 10.1186/s40478-025-01979-0 (PMC11917081; doi:10.1186/s40478-025-01979-0)
Supplement: Supplementary file 1 — Supplementary Material 1 [file 40478_2025_1979_MOESM1_ESM.docx]

**Spatiotemporal perturbations of the plasminogen activation system in a rat model of acute organophosphate intoxication**

**Blackmon, MacMahon et al.**

**SUPPLEMENTARY INFORMATION**

**SUPPLEMENTAL METHODS**

**Method 1: Custom Module workflow**

ImageXpress Micro XLS High Content Analysis System custom module step by step guide for each co-stain:

**PAI/NeuN: % of neurons expressing PAI-1**

1. Find round objects was used to identify DAPI positive nuclei.

2. Find blobs was used to identify NeuN positive neurons.

3. Logical operations were used to identify DAPI positive neurons.

4. Keep marked objects was used to keep the overall shape of the neurons from step 2 but only include neurons that also had DAPI signal.

5. Simple threshold was used to filter out positive PAI-1 signal from background. Positive signal was defined as signal at least twice the intensity of background. In most cases positive signal was at least 3 times higher than background.

6. Grow objects without touching was used on the DAPI positive nuclei to slightly increase their size since PAI-1 could colocalize right outside of the nucleus and usually is found on the cell surface or within the cytoplasm.

7. Logical operations were used to filter out DAPI positive cells that did not express PAI-1.

8. Keep marked objects was used to keep count of the neurons that also had an overlap with PAI-1 positive signal.

9. Measure mask was used to analyze the proportion of NeuN positive neurons that had PAI-1 positive signal.

**PAI/IBA1 and PAI/CD68: % of microglia and phagocytic microglia expressing PAI-1**

1. Find round objects was used to identify DAPI positive nuclei.

2. Find blobs was used to identify CD68 positive cells.

3. Logical operations were used to identify DAPI positive and CD68 positive cells. This is used for the colocalization analysis for the percentage of CD68 positive cells that are co-expressing PAI-1.

4. Keep marked objects using the cells from step 3 and the shapes from step 1 to allow for the entire cell to be marked as CD68 positive.

5. Find round objects was used to identify IBA1 positive cell bodies and some intersecting processes.

6. Find fibers was used to retain microglial processes and mark non-fibrous objects which were the microglial cell bodies.

7. Logical operations were used to retain the entire microglial cell.

8. Keep marked objects was used to keep the overall shape of the microglia, but only include microglia that also had DAPI signal.

9. A filter mask was applied to microglia to individualize each cell so that overlapping cells were limited. For example, without the filter mask, two or more overlapping microglia or microglia that have “connecting” processes would only be counted as one total cell. The filter mask allows us to remove this problem while still allowing the cells to have processes.

10. Simple threshold was used to filter out positive PAI-1 signal from background. Positive signal was defined as signal at least twice the intensity of background. In most cases positive signal was at least 3 times higher than background.

11. Logical operations were used to filter out DAPI positive cells that did not express PAI-1.

12. Logical operations were used to identify IBA1 positive microglia that also expressed PAI-1.

13. Keep marked objects was used to keep the count of microglia that also had an overlap with PAI-1 positive signal.

14. Logical operations were used to identify CD68 positive cells that also expressed PAI-1.

15. Keep marked objects was used to keep the count of CD68 positive cells that also had an overlap with PAI-1 positive signal.

16. Measure mask was used to analyze the proportion of IBA1 positive microglia that had PAI-1 positive signal. It was simultaneously used to analyze the proportion of CD68 positive cells that had PAI-1 positive signal.

**PAI/GFAP: % of astrocytes expressing PAI-1**

1. Find round objects was used to identify DAPI positive nuclei.

2. Find round objects was used to identify GFAP positive cell bodies and some intersecting processes.

3. Logical operations were used to identify DAPI positive astrocytes.

4. Find fibers was used to retain astrocyte processes and mark non-fibrous objects which were the astrocytic cell bodies.

5. Logical operations were used again to retain the entire astrocyte cell.

6. A filter mask was applied to astrocytes to individualize each cell so that overlapping cells were limited. For example, without the filter mask, two or more overlapping astrocytes or astrocytes that have “connecting” processes would only be counted as one total cell. The filter mask allows us to remove this problem while still allowing the cells to have processes.

7. Logical operations were used to include full astrocytes with processes and other astrocytes that did not have processes in the plane but had their cell body in view.

8. Simple threshold was used to filter out positive PAI-1 signal from background. Positive signal was defined as signal at least twice the intensity of background. In most cases positive signal was at least 3 times higher than background.

9. Grow objects without touching was used on the DAPI positive nuclei to slightly increase their size since PAI-1 could colocalize right outside of the nucleus and usually is found on the cell surface or within the cytoplasm.

10. Logical operations were used to filter out DAPI positive cells that did not express PAI-1.

11. Logical operations were used to identify GFAP positive astrocytes that expressed PAI-1.

12. Measure mask was used to analyze the proportion of GFAP positive astrocytes that had PAI-1 positive signal.

**SUPPLEMENTAL TABLES**

**Table S1: Distribution of animals across study endpoints**

| Cohort | Endpoints Evaluated | Tissue used | Sample Size | Figures |
| --- | --- | --- | --- | --- |
| 1  N=38 DFP  N=19 VEH | Seizure behavior  Immunohistochemistry  ELISAs  Plasmin Activity Assay | Coronal Brain Sections  Plasma, Cerebellum,  Cortex, Hippocampus  Plasma | 38/38 DFP, 19/19 VEH  11-12/38 DFP, 11-12/19 VEH  30-36/38 DFP, 14-17/19 VEH  16/38 DFP, 15/19 VEH | 2c  7, 8, 9, 10, 11  5, 6  4 |
| 2 | Transmission Electron Microscopy | Coronal Brain Sections | 2  (1 DFP, 1 VEH) | 3 |

**Table S2: List of primary antibodies used for immunohistochemistry**

| **1° Antibody** | **Host** | **Source** | **Location** | **Product #** | **Lot #** | **Dilution** | **Clonality** | **Clone** | **Subclass** | **RRID** |
| --- | --- | --- | --- | --- | --- | --- | --- | --- | --- | --- |
| Anti-NeuN | Mouse | Millipore | Burlington, MA, USA | MAB377 | 2919676 | 1:500 | Mono | A60 | IgG1 | AB_2298772 |
| Anti-rat Cd68 | Mouse | Bio-Rad | Hercules, CA, USA | MCA341R | 148924C | 1:200 | Mono | ED1 | IgG1 | AB_2291300 |
| Anti-GFAP | Mouse | Cell Signaling | Danvers, MA, USA | 3670 | 6 | 1:1000 | Mono | GA5 | IgG1 | AB_561049 |
| Anti AIF/IBA1 | Goat | Novus | Centennia, CO, USA | NB1001028 | S7C7G2P40 | 1:500 | Poly | N/A | N/A | AB_521594 |
| Anti-PAI-1 | Rabbit | MyBioSource | San Diego, CA, USA | MBS1265040 | F0928A | 1:100 IHC  1:1000 WB | Poly | N/A | IgG | N/A |
| Anti-CD31 | Mouse | Thermo Fisher | Waltham, MA, USA | MA180069 | WB3189522, WB3186081B | 1:50 | Mono | TLD-3A12 | IgG1 | AB_928130 |
| Anti-AQP4 | Mouse | Santa Cruz Biotech | Dallas, TX, USA | SC-32739 |  | 1:50 | Mono | 4/18 | IgG3 | AB_626695 |

**Table S3: List of secondary antibodies used for immunohistochemistry**

| **2° Antibody** | **Host** | **Reacts with** | **Source** | **Product #** | **Lot #** | **Dilution** | **Wavelength** | **Antibody used with** | **RRID** |
| --- | --- | --- | --- | --- | --- | --- | --- | --- | --- |
| Anti-Rabbit IgG (H+L) | Goat | Rabbit | Invitrogen | A21245 | 2299231 | 1:600 | 647 | PAI-1 | AB_2535813 |
| Anti-mouse IgG1 Alexa Fluor | Goat | Mouse | Invitrogen | A21124 | 2300937 | 1:1000 | 568 | GFAP, NeuN | AB_2535766 |
| Anti-mouse AlexaFluor | Goat | Mouse | Invitrogen | A21121 | 2271701 | 1:1000 | 488 | Cd31 | AB_2535764 |
| Anti-goat F(ab)2 | Donkey | Goat | Jackson ImmunoResearch | 705606147 | 144080 | 1:500 | 647 | IBA1 | AB_2340438 |
| Anti-rabbit H+L AlexaFluor | Donkey | Rabbit | Invitrogen | A10042 | 2207536 | 1:600 | 568 | PAI-1 | AB_2534017 |
| Anti-mouse IgG | Donkey | Mouse | Invitrogen | A21202 | 2147618 | 1:1000 | 488 | Cd68 | AB_141607 |
| Anti-rabbit IgG | Goat | Rabbit | Licor | 926-68071 | C80605-15 | 1:10000 | 680 | PAI-1 | AB_10956166 |

**Table S4: RT-qPCR primers**

| **Gene** | **F Primer** | **R Primer** |
| --- | --- | --- |
| *tgfb* | CAAAGACATCACACACAGTA | GGTGTTGAGCCCTTTCCAGG |

The Biorad Assay IDs for the house keeping gene primers are as follows: *actb* (qRnoCID0056984), *hmbs* (qRnoCED0057013), *ywhaz* (qRnoCID0056990).

**Table S5: Normalized Blood plasma concentrations**

| Component Measured | Time point | Group | Average Value | GMR:  DFP vs VEH | CI (95%) | p-value |
| --- | --- | --- | --- | --- | --- | --- |
| **Active Plasmin Content** | 1 DPE | VEH  DFP | 1.56 µM  1.15 µM | 2.09 | 0.3-13.1 | 0.419 |
|  | 3 DPE | VEH  DFP | 0.69 µM  1.17 µM | 1.78 | 0.2-16.6 | 0.6 |
|  | 7 DPE | VEH  DFP | 0.637 µM  0.486 µM | 0.2 | 0.02-1.6 | 0.128 |
|  | 28 DPE | VEH  DFP | 0.41 µM  0.535 µM | 1.41 | 0.2-8.9 | 0.702 |
| **PAI-1 total protein levels** | 1 DPE | VEH  DFP | 0.016 nM  0.149 nM | 6.83 | 3.0-15.4 | <0.001 |
|  | 3 DPE | VEH  DFP | 0.014 nM  0.026 nM | 1.7 | 0.8-3.6 | 0.172 |
|  | 7 DPE | VEH  DFP | 0.015 nM  0.024 nM | 1.2 | 0.5-2.7 | 0.64 |
|  | 28 DPE | VEH  DFP | 0.021 nM  0.022 nM | 0.7 | 0.3-1.6 | 0.406 |

GMR = geometric mean ratio, DFP = diisopropylfluorophosphate intoxicated animals, VEH = vehicle animals, CI = confidence interval, DPE = days post exposure, HIP = hippocampus, COR = cortex, CER = cerebellum

**Table S6: ELISA brain tissue normalized protein concentrations**

| Component Measured | Time point | Brain Region | Group | Average Value (ng/mg protein) | GMR:  DFP vs VEH | CI (95%) | p-value |
| --- | --- | --- | --- | --- | --- | --- | --- |
| **PAI-1 total protein levels** | 1 DPE | HIP  COR  CER | VEH  DFP  VEH  DFP  VEH  DFP | 0.08  2.49  0.08  2.99  0.10  0.28 | 27.7  37.3  3.1 | 20.8-37.0  22.0-63.4  1.4-6.6 | <0.001  <0.001  0.004 |
|  | 3 DPE | HIP  COR  CER | VEH  DFP  VEH  DFP  VEH  DFP | 0.12  0.75  0.12  0.78  0.13  0.36 | 4.4  5.4  2.5 | 2.2-8.7  2.4-12.4  1.0-6.0 | <0.001  <0.001  0.045 |
|  | 7 DPE | HIP  COR  CER | VEH  DFP  VEH  DFP  VEH  DFP | 0.18  0.26  0.1  0.6  0.30  0.18 | 1.7  2.3  0.9 | 0.8-3.7  0.8-7.0  0.3-2.2 | 0.17  0.141  0.788 |
|  | 28 DPE | HIP  COR  CER | VEH  DFP  VEH  DFP  VEH  DFP | 0.11  0.17  0.13  0.07  0.13  0.17 | 1.3  0.5  1.3 | 0.8-2.2  0.3-1.0  0.6-2.7 | 0.214  0.036  0.490 |
| **tPA total protein levels** | 1 DPE | HIP  COR  CER | VEH  DFP  VEH  DFP  VEH  DFP | 0.79  2.15  1.80  2.15  3.74  3.01 | **Only by**  2.2  1.4  0.9 | **region**  1.9-2.6  1.2-1.6  0.8-1.0 | <0.001  <0.001  <0.179 |
|  | 3 DPE | HIP  COR  CER | VEH  DFP  VEH  DFP  VEH  DFP | 0.91  2.49  1.92  2.49  3.32  3.43 |  |  |  |
|  | 7 DPE | HIP  COR  CER | VEH  DFP  VEH  DFP  VEH  DFP | 1.02  2.15  1.89  2.15  3.72  3.26 |  |  |  |
|  | 28 DPE | HIP  COR  CER | VEH  DFP  VEH  DFP  VEH  DFP | 1.01  1.91  2.14  1.91  3.41  3.19 |  |  |  |
| **uPA total protein levels** | 1 DPE | HIP  COR  CER | VEH  DFP  VEH  DFP  VEH  DFP | 0.09  0.49  0.09  0.35  0.15  0.11 | 5.3  4.1  0.7 | 4.1-6.8  2.8-6.2  0.5-0.9 | <0.001  <0.001  0.005 |
|  | 3 DPE | HIP  COR  CER | VEH  DFP  VEH  DFP  VEH  DFP | 0.07  0.25  0.18  0.30  0.11  0.12 | 3.5  1.8  1.1 | 1.5-8.0  0.8-3.8  0.8-1.6 | 0.004  0.139  0.5 |
|  | 7 DPE | HIP  COR  CER | VEH  DFP  VEH  DFP  VEH  DFP | 0.09  0.21  0.18  0.24  0.13  0.16 | 2.0  1.2  1.2 | 1.3-3.2  0.8-1.9  0.9-1.5 | 0.002  0.458  0.277 |
|  | 28 DPE | HIP  COR  CER | VEH  DFP  VEH  DFP  VEH  DFP | 0.09  0.19  0.10  0.16  0.12  0.13 | 1.9  1.6  1.0 | 0.7-5.1  0.9-2.9  0.6-17 | 0.231  0.14  0.901 |

GMR = geometric mean ratio, DFP = diisopropylfluorophosphate intoxicated animals, VEH = vehicle animals, CI = confidence interval, PAI-1 = plasminogen activator inhibitor 1, tPA = tissue-type plasminogen activator, uPA = urokinase plasminogen activator, DPE = days post exposure, HIP = hippocampus, COR = cortex, CER = cerebellum

**Table S7: Percent colocalization of PAI-1 with GFAP positive astrocytes by group, time, and brain region^*^**

|  | VEH | | | | DFP | | | |
| --- | --- | --- | --- | --- | --- | --- | --- | --- |
| Region | 1DPE | 3DPE | 7DPE | 28DPE | 1DPE | 3DPE | 7DPE | 28DPE |
| Amygdala | 25.2 (14.6) | 18.1 (17.5) | 7.6 (3.3) | 21.1 (15.8) | 45.0 (9.8) | 12.5 (9.0) | 7.8 (11.1) | 45.8 (6.4) |
| CA1 | 14.6 (23.5) | 19.9 (2.7) | 10.4 (5.6) | 10.3 (16.0) | 17.0 (14.9) | 13.8 (9.1) | 30.9 (6.7) | 54.7 (10.0) |
| CA3 | 9.6 (13.2) | 5.9 (4.6) | 2.0 (0.2) | 2.9 (4.6) | 14.8 (13.5) | 7.0 (2.5) | 29.0 (20.3) | 48.2 (1.2) |
| Dentate Gyrus | 13.1 (5.1) | 31.8 (5.2) | 12.3 (7.9) | 13.8 (22.2) | 44.8 (11.2) | 9.1 (0.4) | 54.7 (18.2) | 58.6 (6.3) |
| Piriform Cortex | 10.4 (6.7) | 11.3 (4.5) | 5.8 (5.1) | 9.6 (12.1) | 38.1 (16.9) | 20.2 (10.2) | 3.8 (5.3) | 30.2 (27.5) |
| Thalamus | 7.2 (10.6) | 3.8 (1.9) | 1.6 (1.4) | 13.3 (19.2) | 5.7 (4.2) | 1.6 (1.2) | 18.0 (15.5) | 45.9 (26.8) |

^*^ mean (standard deviation)

**Table S8: Percent colocalization of PAI-1 with NeuN positive neurons by group, time, and brain region^*^**

|  | VEH | | | | DFP | | | |
| --- | --- | --- | --- | --- | --- | --- | --- | --- |
| Region | 1DPE | 3DPE | 7DPE | 28DPE | 1DPE | 3DPE | 7DPE | 28DPE |
| Amygdala | 0.2 (0.1) | 0.7 (0.7) | 1.2 (1.3) | 0.5 (0.5) | 4.1 (5.6) | 5.8 (7.0) | 2.6 (3.2) | 9.2 (13.5) |
| CA1 | 2.6 (4.5) | 8.1 (7.1) | 0 (0) | 1.7 (2.9) | 0 (0) | 18.6 (29.9) | 0.6 (0.5) | 2.1 (1.2) |
| CA3 | 0.6 (0.2) | 8.9 (8.7) | 7.0 (11.1) | 12.3 (21.2) | 0.6 (0.5) | 2.1 (2.7) | 0.7 (0.4) | 19.3 (14.7) |
| Dentate Gyrus | 0.4 (0.6) | 1.3 (2.3) | 1.6 (0.9) | 3.9 (6.8) | 7.6 (8.3) | 3.4 (5.9) | 22.9 (24.8) | 1.8 (2.5) |
| Piriform Cortex | 1.3 (2.2) | 0.3 (0.3) | 18.3 (31.3) | 2.0 (0.1) | 16.0 (4.7) | 2.3 (1.8) | 17.0 (23.0) | 19.6 (28.9) |
| Thalamus | 6.3 (8.8) | 0 (0) | 0 (0) | 0.5 (0.6) | 2.9 (4.7) | 0.2 (0.4) | 8.1 (11.9) | 0.9 (1.0) |

^*^ mean (standard deviation)

**Table S9: Percent colocalization of PAI-1 with IBA-1 positive microglia by group, time, and brain region^*^**

|  | VEH | | | | DFP | | | |
| --- | --- | --- | --- | --- | --- | --- | --- | --- |
| Region | 1DPE | 3DPE | 7DPE | 28DPE | 1DPE | 3DPE | 7DPE | 28DPE |
| Amygdala | 0.5 (0.6) | 0.4 (0.4) | 0.6 (0.5) | 0 (0) | 1.1 (1.4) | 6.1 (5.3) | 1.3 (1.8) | 1.7 (2.9) |
| CA1 | 0 (0) | 0.4 (0.7) | 0.6 (1.1) | 2.9 (5.1) | 1.1 (1.5) | 0.2 (0.2) | 9.0 (14.7) | 2.2 (3.0) |
| CA3 | 0 (0) | 0.6 (0.6) | 0.3 (0.5) | 0 (0) | 0 (0) | 0.3 (0.3) | 0.3 (0.5) | 0.1 (0.2) |
| Dentate Gyrus | 1.0 (1.6) | 0.3 (0.4) | 0 (0) | 0 (0) | 0 (0) | 0.5 (0.5) | 0.1 (0.2) | 4.5 (7.5) |
| Piriform Cortex | 0.4 (0.6) | 0.4 (0.7) | 0.2 (0.4) | 1.9 (0.6) | 0.9 (0.7) | 7.8 (8.0) | 6.3 (11.0) | 2.3 (3.3) |
| Thalamus | 0.5 (0.6) | 0 (0) | 1.2 (1.5) | 0.9 (1.0) | 0.2 (0.2) | 0.3 (0.2) | 0.1 (0.3) | 2.5 (3.6) |

^*^ mean (standard deviation)

**Table S10: Percent colocalization of PAI-1 with CD68 positive microglia by group, time, and brain region^*^**

|  | VEH | | | | DFP | | | |
| --- | --- | --- | --- | --- | --- | --- | --- | --- |
| Region | 1DPE | 3DPE | 7DPE | 28DPE | 1DPE | 3DPE | 7DPE | 28DPE |
| Amygdala | 0 (0) | 0 (0) | 0.07 (0.12) | 0 (0) | 0.4 (0.8) | 4.7 (5.5) | 0.6 (1.1) | 0.2 (0.3) |
| CA1 | 0.8 (1.3) | 0 (0) | 0 (0) | 2.0 (3.4) | 0 (0) | 0 (0) | 0 (0) | 0.6 (1.1) |
| CA3 | 0 (0) | 0 (0) | 0 (0) | 0 (0) | 0 (0) | 0 (0) | 0 (0) | 0 (0) |
| Dentate Gyrus | 0 (0) | 0 (0) | 0 (0) | 0 (0) | 0 (0) | 0 (0) | 0 (0) | 1.6 (2.8) |
| Piriform Cortex | 0 (0) | 0 (0) | 0 (0) | 0 (0) | 0 (0) | 2.0 (2.0) | 6.7 (11.6) | 0.1 (0.2) |
| Thalamus | 0 (0) | 0 (0) | 0.2 (0.3) | 0 (0) | 0.2 (0.4) | 0.06 (0.10) | 0.03 (0.06) | 0.2 (0.1) |

^*^ mean (standard deviation)

**Table S11: Housekeeping gene plate from Biorad**

| **Gene** | **PrimePCR Unique Assay ID** |
| --- | --- |
| ***actb^[[1]](#footnote-1)^*** | **qRnoCID0056984** |
| *b2m* | qRnoCED0056999 |
| *camlg* | qRnoCED0009273 |
| *cd163* | qRnoCID0008321 |
| *gapdh* | qRnoCID0057018 |
| ***hmbs*** | **qRnoCED0057013** |
| *hprt1* | qRnoCED0057020 |
| *pgk1* | qRnoCED0002588 |
| *polr2a* | qRnoCED0007537 |
| *ppib* | qRnoCED0006997 |
| *ppox* | qRnoCED0012992 |
| *rpl13a* | qRnoCED0056993 |
| *rps13* | qRnoCED0002931 |
| *rps18* | qRnoCED0003920 |
| *sdha* | qRnoCID0057011 |
| *tubb5* | qRnoCED0053789 |
| ***ywhaz*** | **qRnoCID0056990** |

Bold font denotes housekeeping genes used for

statistical analysis

**SUPPLEMENTAL FIGURES**


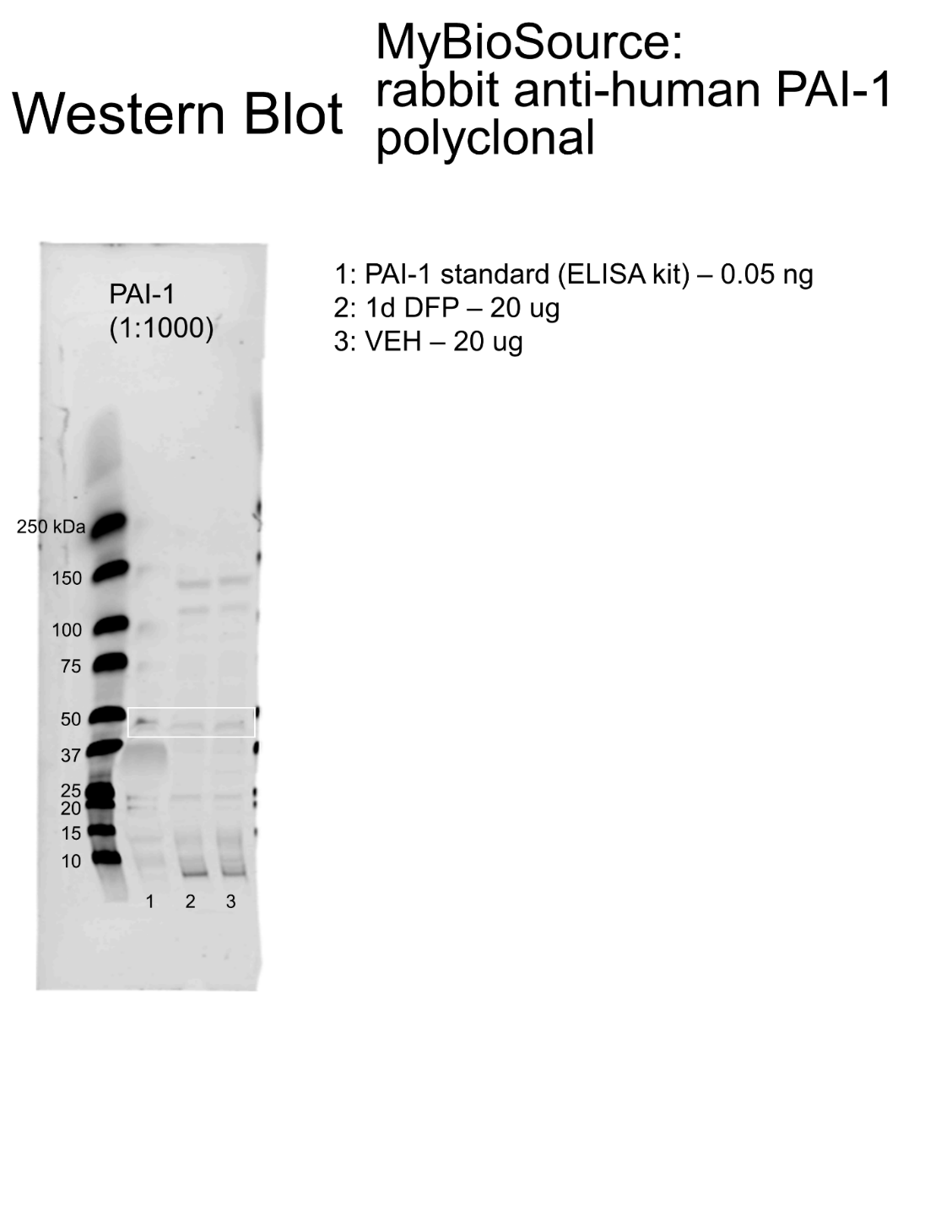


**Fig. S1 Validation of MyBioSource PAI-1 antibody validation** Validation of MyBioSource polyclonal antibody against PAI-1 standard from the Molecular Innovations total PAI-1 ELISA kit. Corresponding band slightly below 50 kDa on lane 1 demonstrates specificity of MyBioSource PAI-1 antibody. No dark bands appear above 50 kDa demonstrating highly specific binding to pure PAI-1 protein. Lanes 2 and 3 are brain extracts from DFP and VEH rats. High molecular weight bands likely correspond to PAI-1 complexes with tPA [1, 2] . Low molecular weight bands likely correspond to PAI-1 reactive center cleavage [3].

**
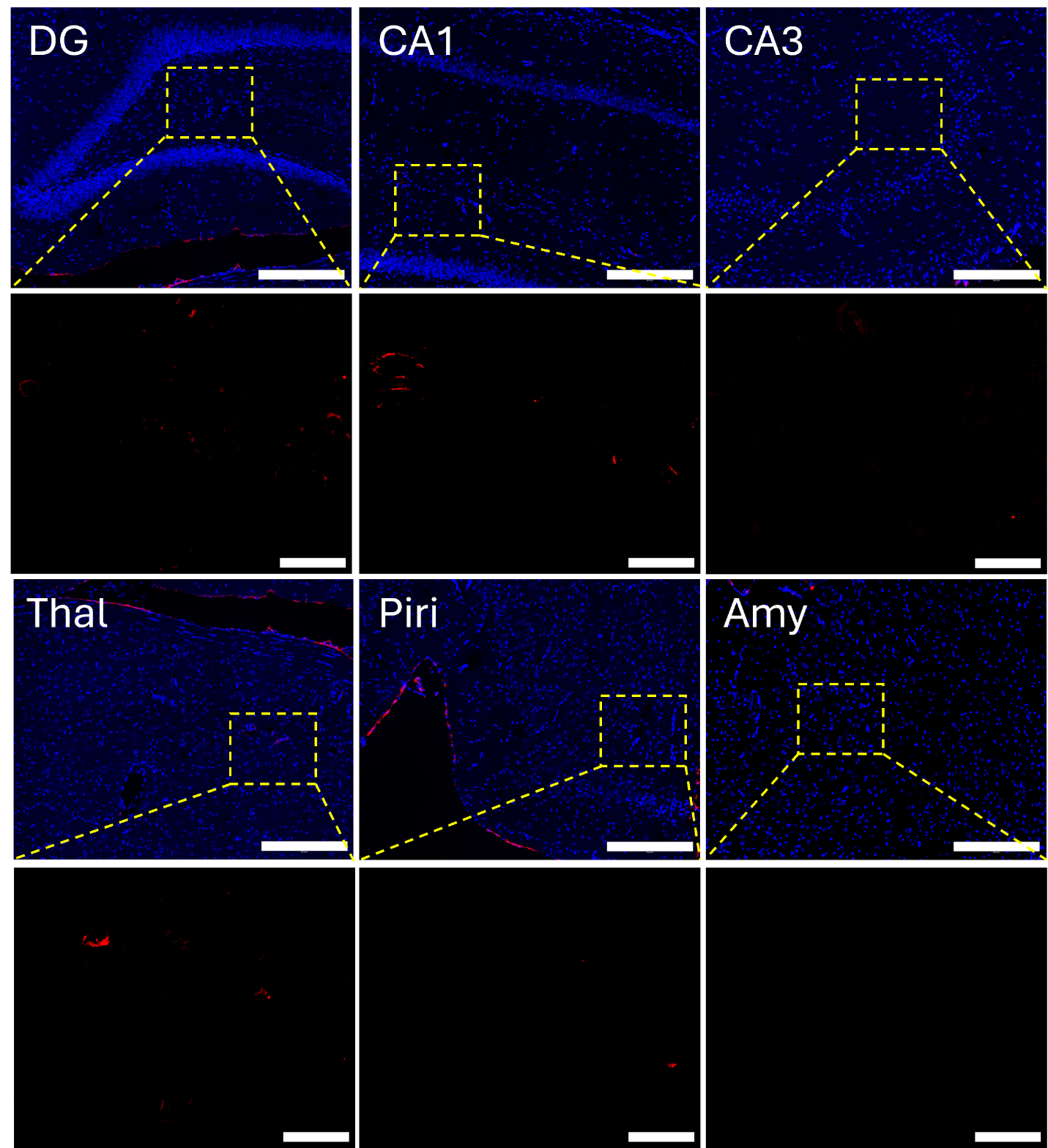
**

**Fig. S2 Vehicle (VEH) animals express negligible PAI-1.** Representative photomicrographs from VEH animals at 28 DPE of PAI-1 (red) immunoreactivity in the dentate gyrus (DG), CA1 and CA3 regions of the hippocampus, thalamus (Thal), piriform cortex (Piri), and amygdala (Amy). Sections were counterstained with DAPI (blue) to identify cell nuclei. Boxed areas are shown at higher magnification in the image below the image with the box (e.g., the second and fourth rows from the top). Bars = 200 µm in low magnification images (first and third rows from the top), and 20 µm in high magnification images (second and fourth rows from the top).


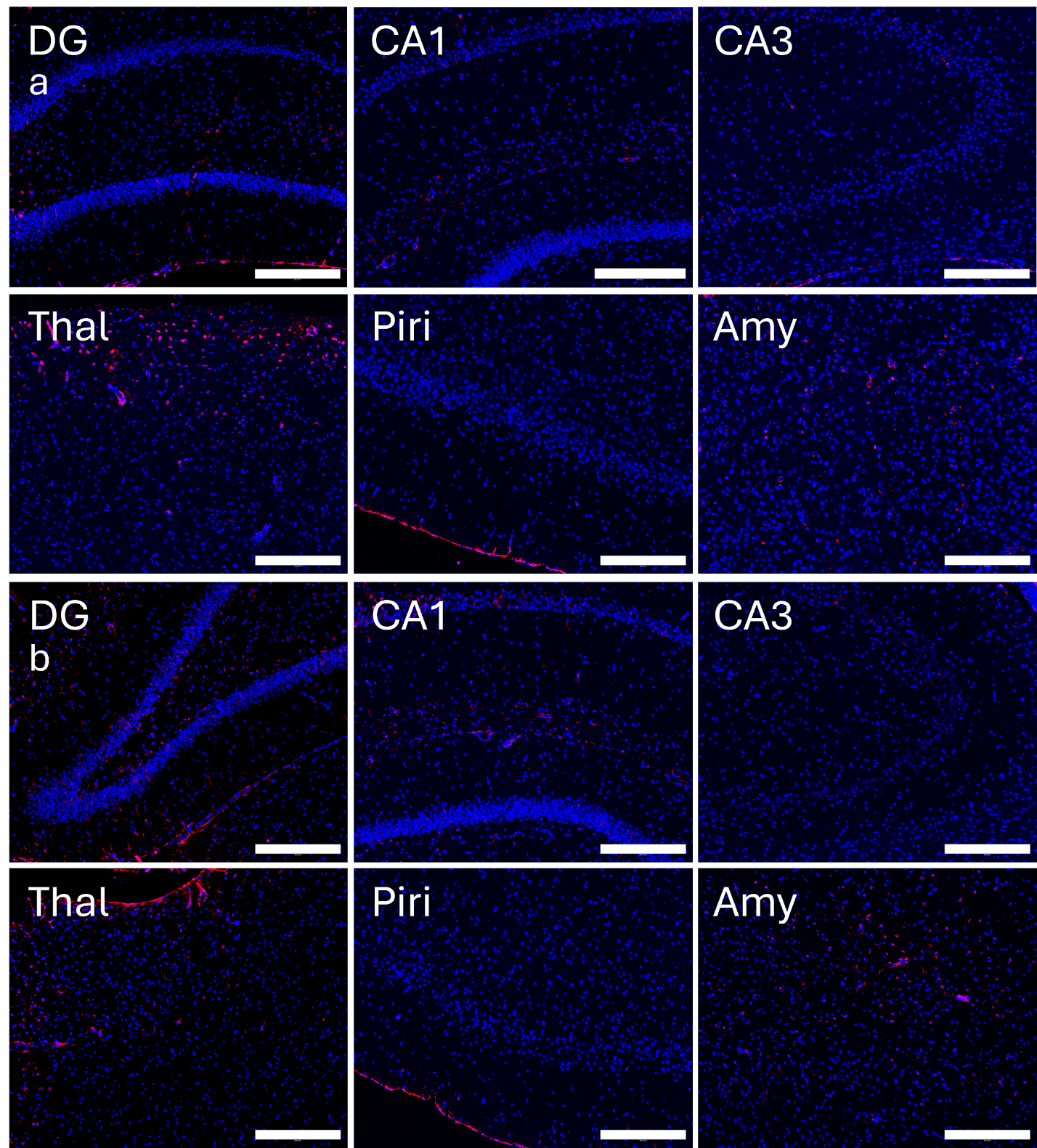


**Fig. S3 Acute DFP intoxication induced weak PAI-1 signaling in multiple brain regions at 1 and 3 DPE.** Representative photomicrographs of PAI-1 (red) immunoreactivity in the dentate gyrus (DG), CA1 and CA3 regions of the hippocampus, thalamus (Thal), piriform cortex (Piri), and amygdala (Amy) at 1 (**a**) and 3 (**b**) DPE. Sections were counterstained with DAPI (blue) to identify cell nuclei. Bars = 200 µm

**
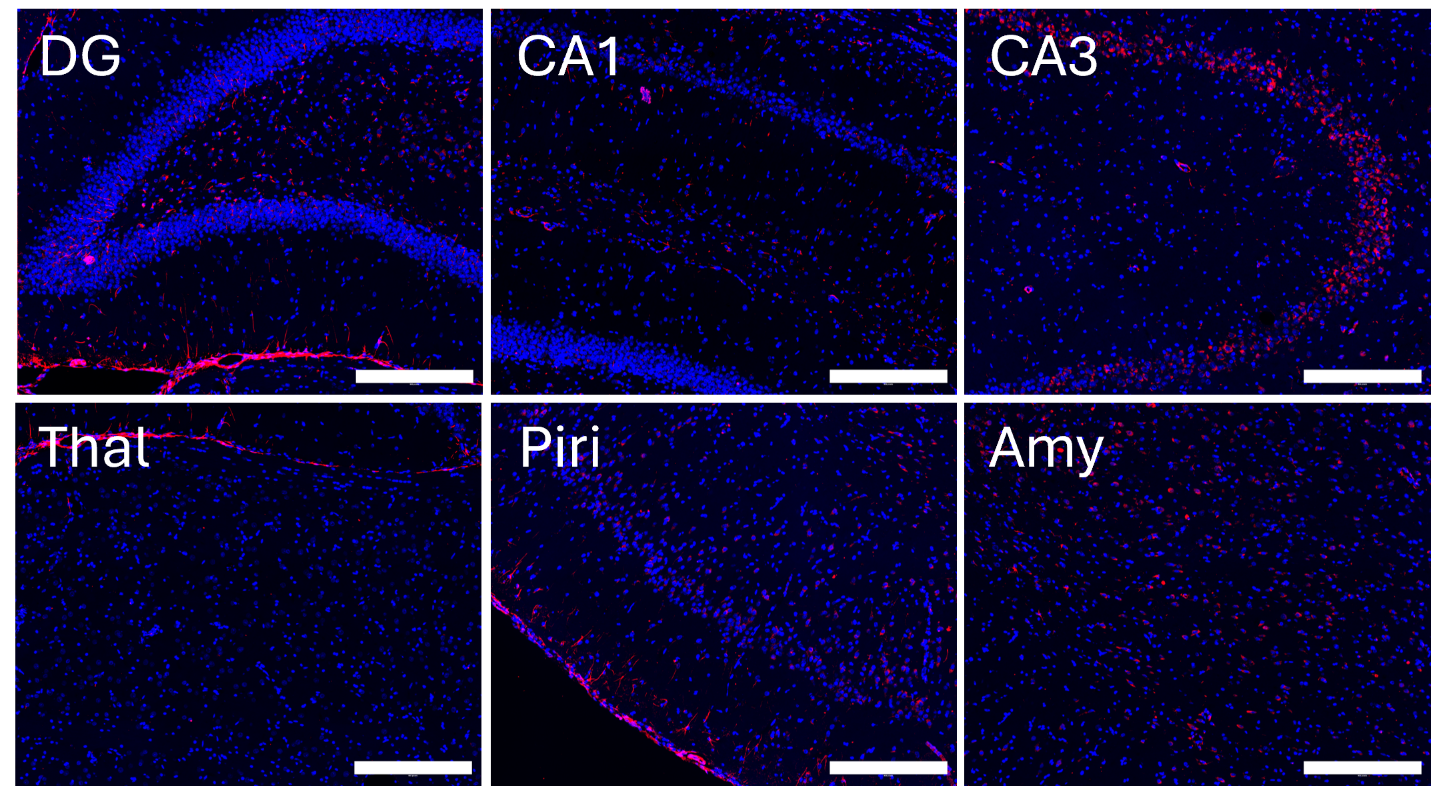
**

**Fig. S4 Acute DFP intoxication induced PAI-1 expression in multiple brain regions at 7 DPE.** Representative photomicrographs of PAI-1 (red) immunoreactivity in the dentate gyrus (DG), CA1 and CA3 regions of the hippocampus, thalamus (Thal), piriform cortex (Piri), and amygdala (Amy) at 7 DPE. Sections were counterstained with DAPI (blue) to identify cell nuclei. Bars = 200 µm.

**
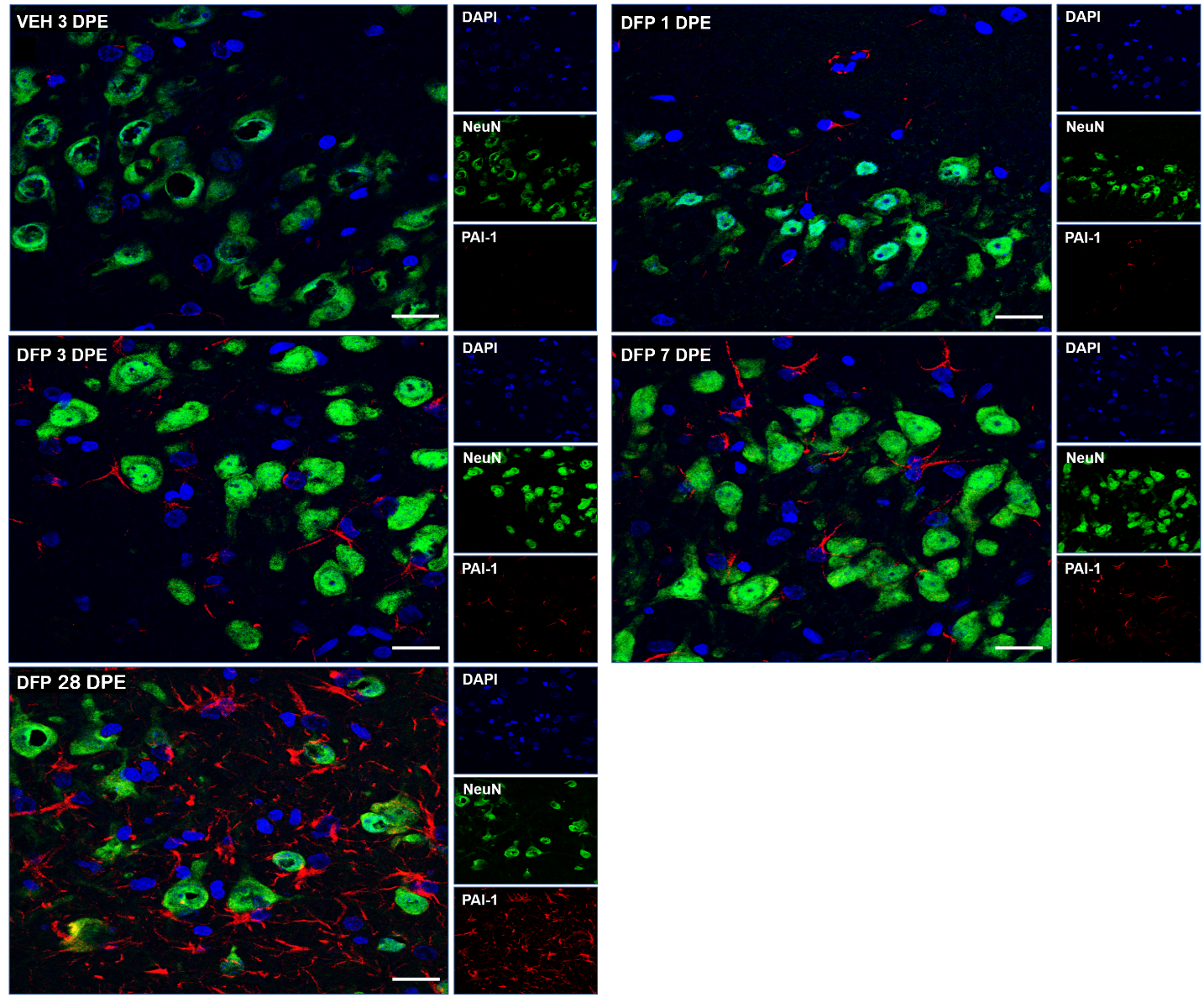
**

**Fig. S5** **DFP-induced PAI-1 expression overlays but does not co-localize to neurons. (a)** Representative photomicrographs of the hilus of the hippocampus at 1, 3, 7 and 28 DPE of a VEH and DFP animal immunostained for PAI-1 (red) and NeuN (green). Sections were counterstained with DAPI to identify cell nuclei. Bar = 20 µm.


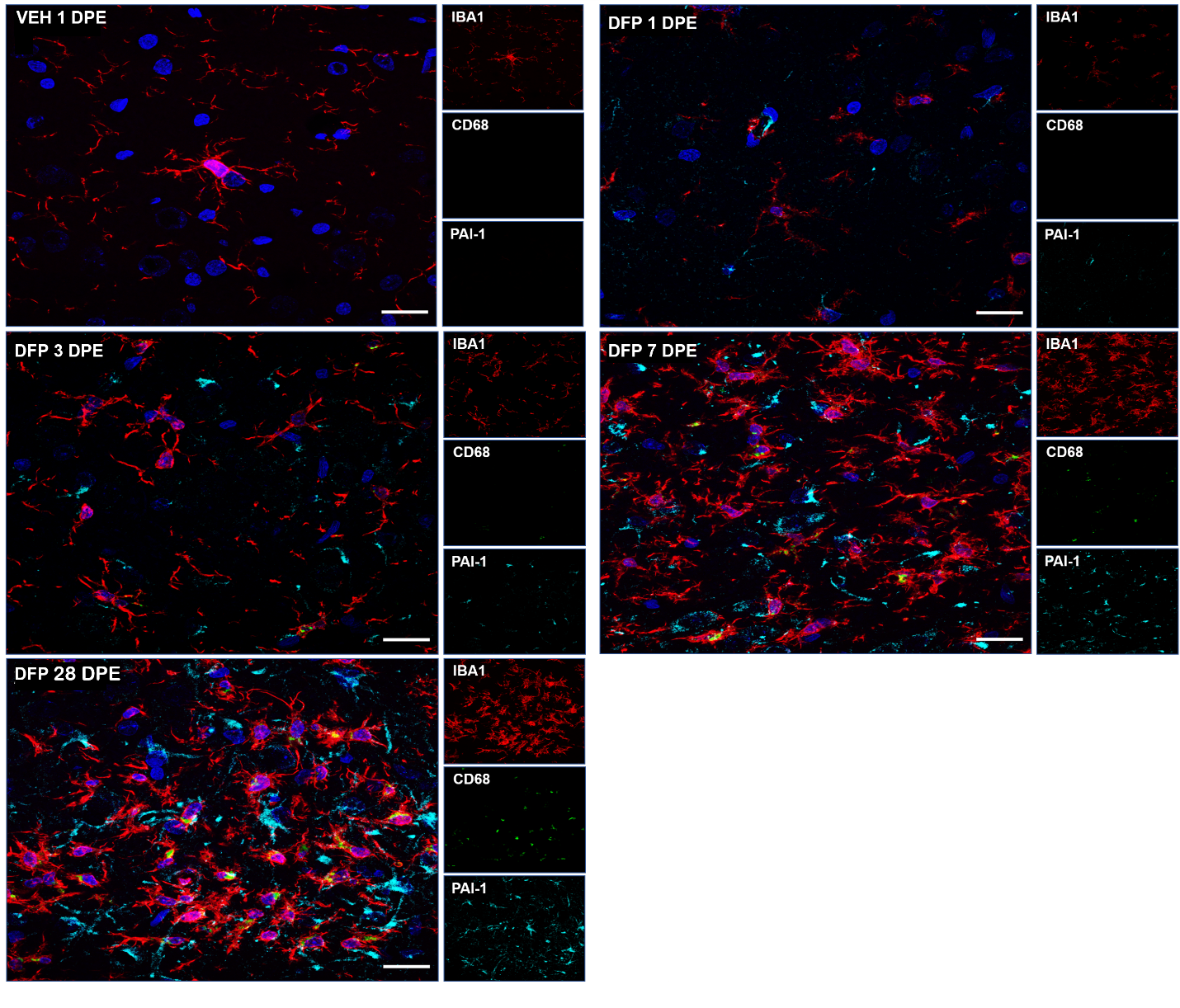


**Fig. S6 DFP-induced PAI-1 expression was minimally co-expressed in CD68+ and in IBA1+ microglial cells following DFP intoxication (a)** Representative photomicrographs of the hilus of the hippocampus from VEH and DFP animals immunostained for IBA1 (red), CD68 (green), and PAI-1 (cyan) at 1, 3, 7 and 28 DPE. Sections were counterstained with DAPI (blue) to identify cell nuclei. Bar = 20 µm.

**REFERENCES**

1. Björquist, P., et al., *Plasminogen activator inhibitor type-1 interacts exclusively with the proteinase domain of tissue plasminogen activator.* Biochimica et Biophysica Acta (BBA) - Protein Structure and Molecular Enzymology, 1994. **1209**(2): p. 191-202.

2. Alessi, M.C., et al., *Molecular forms of plasminogen activator inhibitor-1 (PAI-1) and tissue-type plasminogen activator (t-PA) in human plasma.* Thromb Res, 1991. **62**(4): p. 275-85.

3. Eren, M., et al., *PAI-1 is a critical regulator of FGF23 homeostasis.* Science Advances, 2017. **3**(9): p. e1603259.

1. [↑](#footnote-ref-1)
